# Genome-wide analysis of genomic alterations induced by oxidative DNA damage in yeast

**DOI:** 10.1093/nar/gkz027

**Published:** 2019-01-22

**Authors:** Ke Zhang, Dao-Qiong Zheng, Yang Sui, Lei Qi, Thomas D Petes

**Affiliations:** 1 College of Life Science, Zhejiang University, Hangzhou 310058, China; 2 Ocean College, Zhejiang University, Zhoushan 316021, China; 3 Department of Molecular Genetics and Microbiology, Duke University School of Medicine, Durham, NC 27710, USA

## Abstract

Oxidative DNA damage is a threat to genome stability. Using a genetic system in yeast that allows detection of mitotic recombination, we found that the frequency of crossovers is greatly elevated when cells are treated with hydrogen peroxide (H_2_O_2_). Using a combination of microarray analysis and genomic sequencing, we mapped the breakpoints of mitotic recombination events and other chromosome rearrangements at a resolution of about 1 kb. Gene conversions and crossovers were the two most common types of events, but we also observed deletions, duplications, and chromosome aneuploidy. In addition, H_2_O_2_-treated cells had elevated rates of point mutations (particularly A to T/T to A and C to G/G to C transversions) and small insertions/deletions (in/dels). In cells that underwent multiple rounds of H_2_O_2_ treatments, we identified a genetic alteration that resulted in improved H_2_O_2_ tolerance by amplification of the *CTT1* gene that encodes cytosolic catalase T. Lastly, we showed that cells grown in the absence of oxygen have reduced levels of recombination. This study provided multiple novel insights into how oxidative stress affects genomic instability and phenotypic evolution in aerobic cells.

## INTRODUCTION

Reactive oxidative species (ROS), including ·O_2_, H_2_O_2_ and ·OH, are produced within eukaryotic cells, largely as a consequence of electron transport in the mitochondria during aerobic growth (1). The intracellular levels of ROS are normally low due to antioxidative systems: both small antioxidant molecules such as glutathione and ascorbic acid, and enzymatic systems such as superoxide dismutases and catalases (2). Although the levels of ROS are low under normal growth conditions, exposure of cells to certain environmental conditions including ultraviolet light (3), heat shock (4), certain pathogens (5) and several types of chemicals (6) lead to oxidative stress and damage to multiple species of biological macromolecules.

Hydrogen peroxide (H_2_O_2_) is one of the oxidizing compounds that has been studied most extensively. In an interaction with iron, H_2_O_2_ forms ·OH and ^–^OH, and these oxidants are likely to be main DNA-damaging agents (7). Oxidative damage results in more than 80 different types of base damage, as well as single-strand nicks and double-strand breaks (DSBs) (7). Both single-strand nicks and DSBs stimulate mitotic recombination (8). In a previous study, Brennan *et al.* (9) showed that H_2_O_2_ treatment of yeast stimulated mitotic gene conversion between *ade2* heteroalleles. In a study of H_2_O_2_-induced genomic alterations on chromosome V, Hayashi and Umezu (10) showed a dose-dependent elevation of chromosome loss, crossovers, and gene conversion events by H_2_O_2_.

In our study, we use DNA microarrays and DNA sequencing to map H_2_O_2_-induced events throughout the yeast genome. We show that H_2_O_2_-treatment of yeast results in very high levels of mitotic recombination and other genomic alterations including mutations. We also found that yeast cells grown anaerobically or in the presence of the ROS-scavenger glutathione had reduced levels of spontaneous recombination compared to cells grown aerobically. Our analysis demonstrated the potent and diverse mechanisms of oxidative DNA damage in the eukaryotic genome.

## MATERIALS AND METHODS

### Strain construction and medium

The genotypes of yeast strains are given in Supplementary Table S1. Details about strain construction are provided in SI Text (Supplementary data), and the primers used in constructions and analyses are in Supplementary Table S2. Growth medium, and genetic procedures were standard.

### SNP microarray analysis

Analysis of genomic alterations using microarrays was done as described previously (11,12). In brief, genomic DNA from the experimental strain was isolated and labeled with Cy5-dUTP, and control DNA from the fully heterozygous strain JSC24-2 (12) was labeled with Cy3-dUTP. The samples were mixed and hybridized to the SNP microarrays at 62°C. The ratio of hybridization of the two differentially labeled samples was examined using the GenePix scanner and GenePix Pro-6.1 software. Ratios of hybridization for each oligonucleotide were normalized to the Cy5/Cy3 ratio of all of the oligonucleotides on the microarray. We examined the hybridization ratios initially in a window of nine SNPs moved one SNP at a time using R script. Subsequent analysis of break points was done at single-SNP resolution. The principles distinguishing homozygous and heterozygous SNPs were described previously (11,12). The sequences of the oligonucleotides in the arrays and the designs of the arrays are on the Gene Expression Omnibus (GEO) Website (https://www.ncbi.nlm.nih.gov/geo/) at the addresses: GPL20144 (whole-genome) and GPL21552 (chromosome IV-specific).

### CHEF gel electrophoresis and Southern analysis

Yeast cells were embedded in plugs made of 0.8% low-melt agarose and DNA was released *in situ* as described in (13). The CHEF gel electrophoresis was carried out using a Biorad CHEF Mapper system as described in (13). Following electrophoresis, the DNA was transferred to nylon membranes. Hybridization probes were prepared by PCR amplification of yeast genomic DNA using a commercial Digoxigenin-dUTP DNA labeling kit (Roche). Details of the hybridization conditions were described in our previous study (14).

### Whole-genome sequencing

DNA samples of yeast strains were extracted using EZNA yeast DNA kit (Omega, Doraville, USA) and sheared by sonication to fragments of about 400 bp. Whole-genome sequencing was performed on the Illumina HiSeq 2500 sequencer using a 2 × 150-bp paired-end indexing protocol. The BWA software was used to align the high-quality reads to the sequence of the S288c genome (15). Base substitutions and small in/dels were detected using Samtools (16) and VarScan (17).

### Determining the rate of spontaneous crossovers of the right arm of chromosome IV

These experiments employed a diploid strain (DZ5) that was heterozygous for an insertion of *URA3* near the right telomere of IV. Heterozygous strains are sensitive to 5-FOA. Derivatives that are 5-FOA^R^ are usually a consequence of a recombination event on the right arm of chromosome IV or chromosome loss. Since chromosome loss results in loss of heterozygosity (LOH) for markers on both arms of IV, these possibilities can be distinguished by examining LOH for polymorphisms located on the left arm of IV. Relative to W303-1A, the YJM789-derived homolog has a 100 bp deletion located near coordinate 435,000 on the left arm. Using PCR with primers flanking this sequence (vChrIVMonS and vChrIVMonA, Supplementary Table S2) followed by gel electrophoresis, we could readily distinguish whether the 5-FOA^R^ derivatives retained heterozygosity. Yeast cells were plated on YPD solid medium to form single colonies under both aerobic and anaerobic conditions for 72 h. Fifteen colonies of each strain were suspended in sterile water and then plated on 20 5-FOA-containing plates. Experiments were repeated three times. The 5-FOA^R^ colonies were calculated, and the frequency of crossovers was converted into rates using the method of the median (18).

## RESULTS

Previous studies of the genome-destabilizing effects of the oxidizing agent H_2_O_2_ have usually been restricted to analyzing these effects on one cellular process (for example, mutations) at one genetic locus. Below, we use microarray analysis and genomic sequencing to characterize the effects of H_2_O_2_ on a number of different types of changes throughout the yeast genome. First, we examined the effects of acute exposure of G_1_-synchronized yeast cells to H_2_O_2_ on the rates of mitotic crossovers, gene conversion events, large deletions/duplications, and ploidy changes; both selected events on chromosome IV and unselected events throughout the genome were mapped. Second, we physically monitored DSBs produced by H_2_O_2_ treatment of G_1_-arrested cells. Third, we mapped recombination events in cells exposed to multiple cycles of treatment with H_2_O_2_. Fourth, to look for the frequency of H_2_O_2_-induced point mutations, we sequenced the genomes of five of the isolates that were treated with 20 cycles of H_2_O_2_. Fifth, we characterized an amplification of a chromosomal region that occurred in a sub-cultured isolate, demonstrating that amplification of *CTT1* was responsible for the increased resistance to H_2_O_2_. Lastly, we demonstrated the yeast cells grown anaerobically have a reduced rate of recombination compared to aerobically-grown cells. Each of these findings will be discussed below.

### Genomic alterations associated with H_2_O_2_-treated G_1_-synchronized cells

#### Dose-dependent elevation in the rate of mitotic crossovers stimulated by H_2_O_2_

**Figure 1.**
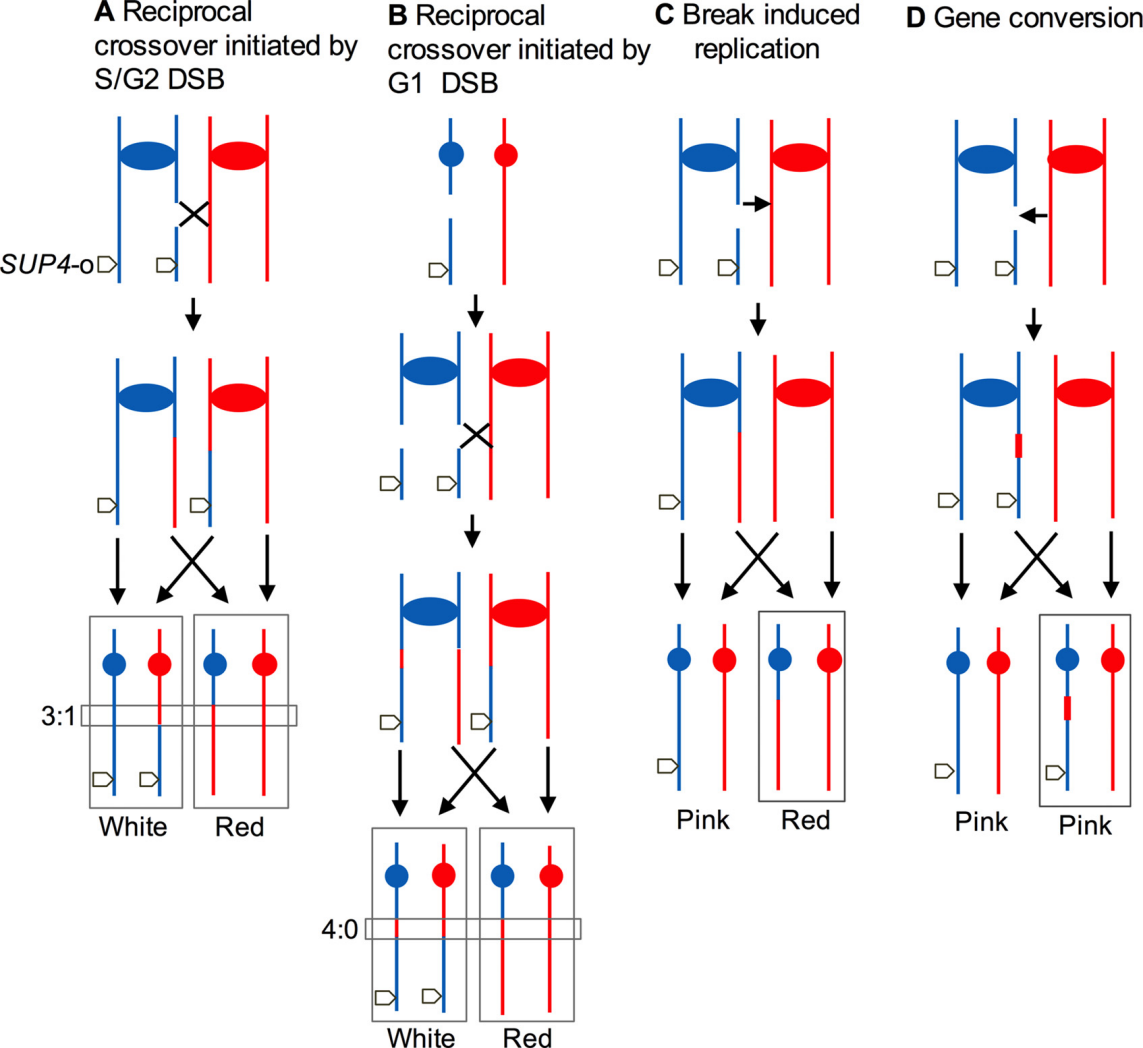
Patterns of mitotic recombination. In A–D, we show genetic exchanges in a diploid heterozygous for an insertion of *SUP4-o* located near the right end of chromosome IV. Red and blue lines indicate the homologs derived from the haploid parental strains W303-1A and YJM789, respectively; the centromeres are shown as ovals and circles. The horizontal rectangles indicate gene conversion tracts; vertical rectangles enclose the daughter cells with recombinant chromosomes. (**A**) Reciprocal crossover event induced by breaking one chromatid in an S- or G_2_-phase cell. A 3:1 conversion tract was associated with the crossover. Segregation of the chromatids as indicated by the arrows would produce one cell homozygous for *SUP4-o* and one cell lacking *SUP4-o* which would give rise to white and red sectors, respectively. (**B**) In this figure, the recombination event is initiated by a DSB on an unreplicated chromosome. Replication of the broken chromosome produces two chromatids broken at the same position, and repair of the resulting breaks generates a 4:0 conversion event associated with the crossover. (**C**) Recombination by break-induced replication. Following a DSB formed on one blue chromatid, the centromere-distal part is lost and the centromere-proximal end invades a red chromatid. The subsequent replication event results in a large non-reciprocal LOH region. (**D**) Gene conversion unassociated with a crossover.

We first measured the effect of H_2_O_2_ on the rate of mitotic crossover in a one Mb-interval on chromosome IV that represents about 10% of the genome. The strain used in most of our studies is JSC25-1, a diploid created by mating two sequence-diverged haploids W303-1A and YJM789 (12). Near the right end of chromosome IV on the YJM789-derived homolog (shown in blue in Figure 1), the strain has an insertion of *SUP4-o*, a tRNA gene encoding an ochre suppressor. The diploid is also homozygous for the *ade2-1* ochre mutation. Diploids with this mutation and with zero, one or two copies of *SUP4-o* form red, pink or white colonies, respectively (12). Details of the strain genotypes and constructions are given in Tables S1 and S2.

As shown in Figure 1A and B, a crossover between *SUP4-o* and the centromere can result in a reciprocal LOH in the daughter cells, producing a red/white sectored colony. Crossovers are associated with the formation of heteroduplexes (regions of DNA composed of strands derived from different molecules), and repair of mismatches in the heteroduplex can result in the non-reciprocal transfer of information from one homolog to the other (8). These events (gene conversions) are shown within narrow horizontal rectangles in Figure 1; vertical rectangles enclose the chromosomes of the daughter cells containing the recombinant chromosomes. In Figure 1A, we show that a DSB in one chromatid results in a conversion event in which three chromatids have information derived from the ‘red’ chromatid and one chromatid with ‘blue’ information.

Surprisingly, in several previous studies of mitotic recombination in yeast (12,19), we showed that sectored colonies were often observed in which all four chromosomes contained a region derived from one of the parental chromosomes (outlined with a horizontal rectangle in Figure 1B); such events were termed 4:0 conversion events. One mechanism by which 4:0 conversion events can be explained is by formation of a DSB in G_1_, followed by replication of the broken chromosome to yield two chromatids broken at the same position. If one of the chromatids is repaired by a conversion event associated with a crossover and the other repaired by a conversion event unassociated with a crossover, the pattern shown in Figure 1B would result. Several arguments support this model. First, although DSBs in some stages of the cell cycle cause arrest until the break is repaired, the G_1_ checkpoint is weak. Even with a dose of radiation that kills more than 90% of G_1_-synchronized cells, almost all of the irradiated cells progress into S-phase (20). Second, when G_1_-synchronized yeast cells are treated with ionizing radiation, 4:0 conversion events are observed; G_2_-irradiated cells produce 3:1 conversion events (21). Third, resection of broken ends (a necessary intermediate in homologous recombination) is inefficient in G_1_ cells (22). Fourth, the frequency of 4:0 conversion events is much too high to represent two independent 3:1 conversion events for both spontaneous and induced events (11,12). Lastly, Esposito (23) found that heteroallelic recombination events in yeast had recombination patterns consistent with G_1_-initiated events.

In addition to crossovers, DSBs can be repaired by the non-reciprocal process of break-induced replication (BIR) (8). BIR events lead to pink/red sectors rather than red/white sectors (Figure 1C) and are infrequent relative to crossovers in wild-type strains (24). Lastly, gene conversion events that are unassociated with crossovers do not generate a sectored colony (Figure 1D), but can be detected by non-selective methods to be described below.

**Figure 2.**
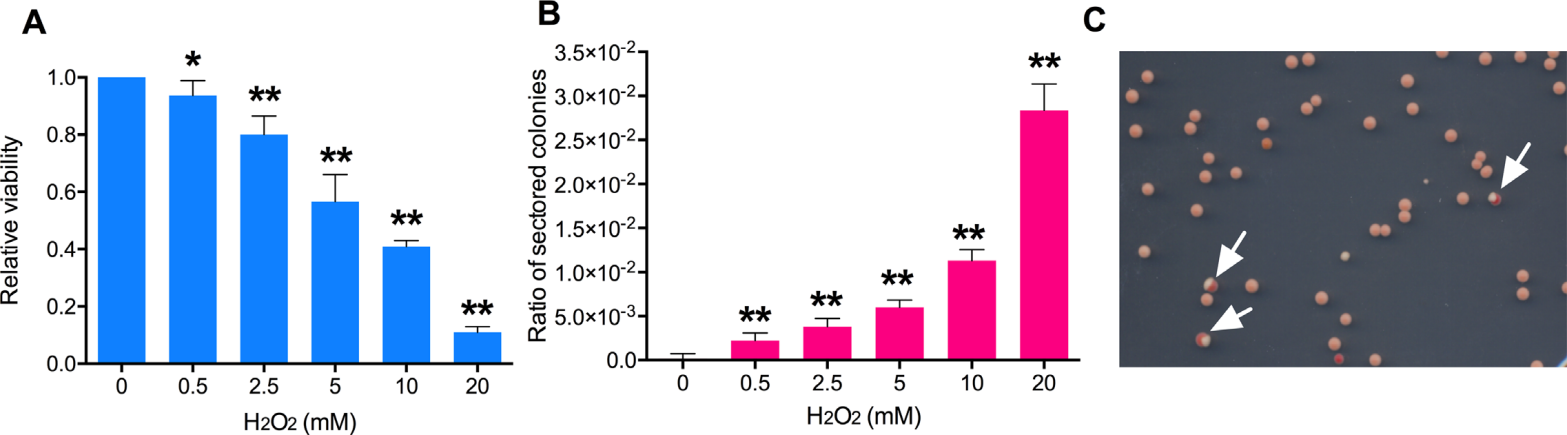
Exposure to H_2_O_2_ causes loss of cell viability and elevated mitotic recombination. Experiments were performed three times, and the error bars for the combined experiments represent 95% confidence limits. All values were compared to the values for the untreated strain by *t*-tests, and significant differences with the untreated strain are shown by single asterisks (*P* ≤.05) or double asterisks (*P* ≤.01). (**A**) Cell viability of yeast strain JSC25-1 after exposure to H_2_O_2_ (0.5–20 mM) treatment. Viability is shown relative to an untreated strain. (**B**) The frequency of crossovers as assayed by the frequency of sectored red/white colonies formed on YPD plates after one-hour treatment with H_2_O_2_. The ‘ratio of sectored colonies’ on the Y-axis is the number of sectored colonies divided by the total colonies. (**C**) An example of colonies derived from cells treated with 20 mM H_2_O_2_. Arrows show sectored colonies.

Before mapping LOH regions, we measured the frequency of red/white sectored colonies as a function of the dose of H_2_O_2_. For these experiments, JSC25-1 cells were collected at logarithmic phase, and synchronized in G_1_ phase using the α-pheromone; JSC25-1 has a deletion of the *MATα*, rendering it sensitive to the reversible arrest caused by the α-pheromone (12). The G_1_-synchronized cells were treated for one hour with concentrations of H_2_O_2_ between 0.5 and 20 mM. The cells were then plated on solid medium and allowed to form colonies. With the increasing concentrations of H_2_O_2_, we observed a loss in cell viability (Figure 2A), but an increase in sectored colonies (Figure 2B). In the absence of H_2_O_2_, the frequency of sectored colonies in untreated JSC25-1 cells is 3.1 × 10^−5^/division in the 1.1 Mb interval between *CEN4* and the *SUP4*-o marker. Relative to this frequency, H_2_O_2_ treatment stimulated sector formation by factors of 72 (0.5 mM), 122 (2.5 mM), 194 (10 mM), 374 (15 mM) and 914 (20 mM). It should be noted that, since only half of crossover events have a pattern of segregation that produces LOH, the rate of crossovers is twice the frequency of sectored colonies (12).

It is possible that a sector could be formed artifactually if two cells (one capable of producing a white colony and one capable of producing a red colony) were located next to one another on the plate. To exclude this possibility, we micromanipulated individual H_2_O_2_-treated (10 mM) cells to specific positions on plates and allowed them to form colonies. Of 1484 colonies derived from such cells, 16 (1%) formed red/white sectored colonies. This frequency is similar to that observed in Figure 2B. In summary, our observations suggest that H_2_O_2_ very strongly stimulates mitotic recombination of yeast even at the concentrations at which yeast cells maintain substantial (>10%) viability.

#### Mapping of genomic alterations induced by H_2_O_2_ in G_1_-synchronized cells

The sectored colonies generated as described above were analyzed using either microarrays designed to detect LOH events on the right arm of chromosome IV (12) or microarrays designed to examine LOH throughout the genome (11). The two types of microarrays detect LOH for 2300 SNPs on the right arm of IV and 15 000 SNPs distributed throughout the genome, respectively. On these arrays (termed ‘SNP arrays’), each SNP is represented by four 25-base oligonucleotides, two identical to the Watson and Crick sequences of the W303-1A version of the SNP and two identical to the Watson and Crick sequences of the YJM789 version. If the diploid is heterozygous for the SNP, the relative amounts of hybridization to all four oligonucleotides are similar. LOH for the assayed SNP results in a higher level of hybridization to the oligonucleotides representing one allele, and a reduced level of hybridization to the oligonucleotide representing the other allele (25). Relative levels of hybridization are measured by labeling the experimental genomic DNA with one fluorescent nucleotide (Cy5) and genomic DNA from a fully-heterozygous diploid control strain with a different label (Cy3) (12). The samples are mixed, hybridized to the microarray, and the resulting levels of hybridization to each fluorescent probe are determined using a GenePix scanner.

**Figure 3.**
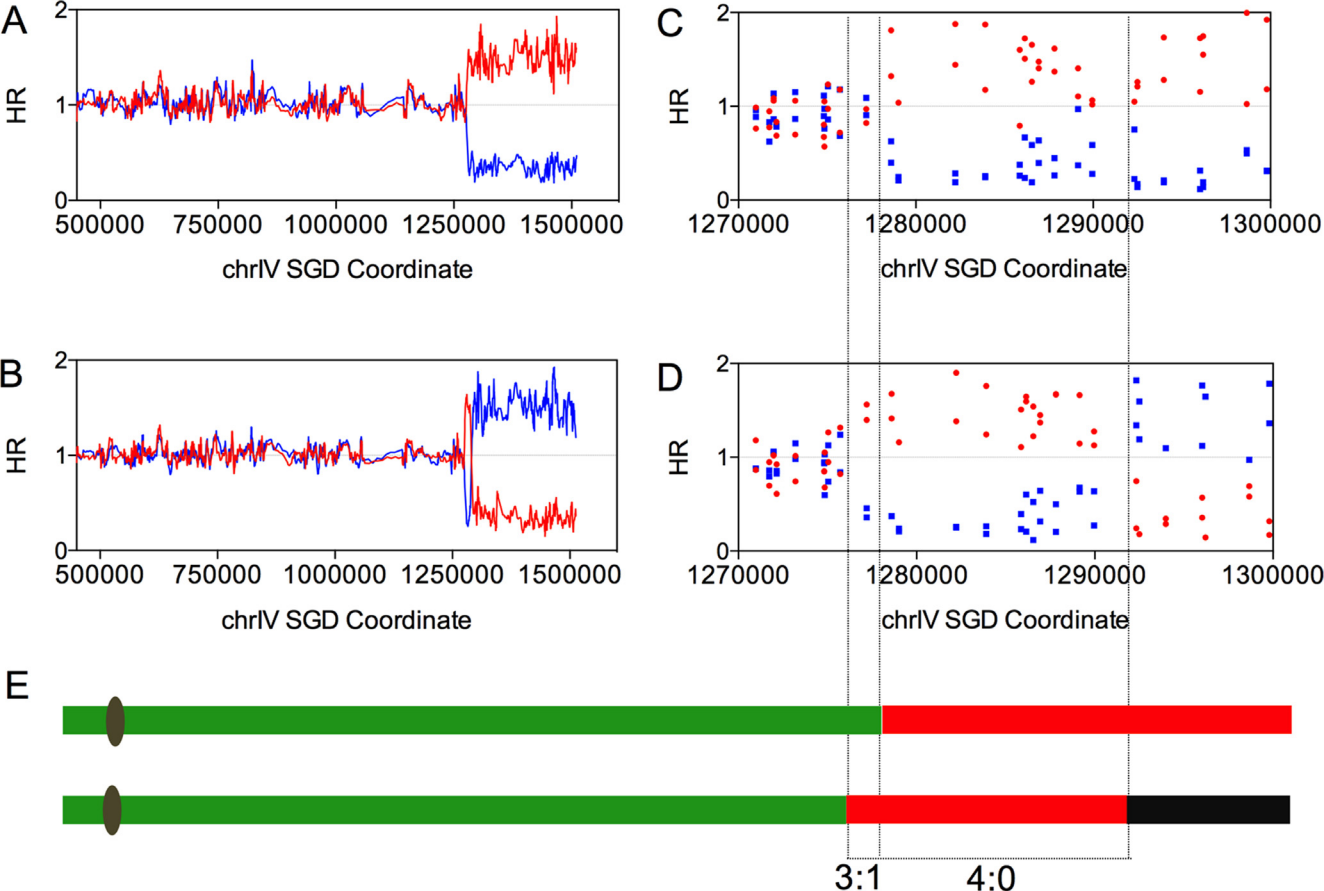
Mapping of a crossover with an associated conversion event by SNP microarrays. The values on the Y-axis show the normalized hybridization ratio (HR) of genomic DNA to oligonucleotides that are specific to SNPs from the W303-1A and YJM789 backgrounds. The values on the X-axis indicate the SGD coordinates of the SNPs along chromosome IV. The hybridization ratio values about 0.3, 1, 1.5 represent zero, one, and two copies of W303-1A- (red points or lines) or YJM789- (blue points or lines) derived homolog. (**A**) Low-resolution depiction of a reciprocal crossover analyzed by the SNP microarrays in the red sector. (**B**) Low-resolution depiction of a reciprocal crossover analyzed by the SNP microarrays in the white sector. (**C**) High-resolution depiction of the reciprocal crossover shown in A. (**D**) High-resolution depiction of the reciprocal crossover shown in B. (**E**) Schematic depiction of the crossover event. The upper and lower lines represent the red and white sectors, respectively. The green, red, and black segments indicate heterozygous SNPs, homozygous for W303-1A-specific SNPs, and homozygous for YJM789-specific SNPs, respectively. The region between three short dashed lines shows a 3:1/4:0 conversion tract associated with this crossover event.

We performed this type of analysis on both red and white sectors of 73 sectored colonies derived from G_1_-synchronized cells exposed to H_2_O_2_. Of these 73 colonies, 45 were from cells treated with 20 mM H_2_O_2_ and 28 were from cells treated with 0.5 mM. Figure 3A and B shows the SNP microarray analysis of the red and white sectors, respectively, of one of these colonies (HO-20-22). Red and blue lines indicate the hybridization ratio (relative to the control heterozygous strain) to W303-1A- and YJM789-derived SNPs, respectively. At low resolution, it is apparent that the crossover occurred near SGD (Saccharomyces Genome Database) coordinate 1250 kb on chromosome IV. At higher resolution (Figure 3C and D), we found that there is a single LOH transition in the red sector between coordinates 1277206 and 1278958, and two transitions in the white sector between coordinates 1275710–1277206 and 1289953–1292325. We show three parallel lines extending between Figure 3C, D and E that define the borders of the conversion event. In the segment defined by the left two lines, in the red sector (Figure 3C) the hybridization values for the SNPs are have a value of ∼1, indicating that these SNPs are heterozygous. In the comparable position in the white sector (Figure 3D), the SNPs derived from the red chromosome have a hybridization ratio of ∼1.6, whereas the SNPs derived from the blue chromosome have a hybridization ratio of about 0.2. This pattern indicates that the SNPs derived from the red chromosome are homozygous, present on both homologs in the white sector. Thus, considering both sectors within this segment, the red SNPs are represented three times and the blue SNPs are represented once, indicating a 3:1 conversion. In the segment defined by the right two parallel lines, both sectors are homozygous for the SNPs derived from the red homolog, indicating a 4:0 conversion. Conversion events in which a 3:1 region is adjacent to a 4:0 region are consistent with the repair of two broken chromatids in which the repair-associated conversion tracts are of different lengths (12). A pattern of heteroduplex formation and mismatch repair that could have generated the LOH patterns shown in Figure 3 is shown in Supplementary Figure S1. Distal to the region of conversion, the red sector is homozygous for red SNPs, and the white sector is homozygous for blue SNPs, as expected for a reciprocal crossover.

In Figure 3E, we depict the LOH events of the HO-20-22 sectored colony as a pair of lines. The upper line represents the patterns of LOH in the red sector with the green and red regions indicating heterozygous SNPs and SNPs homozygous for the W303-1A-derived alleles, respectively. In the lower line of Figure 3E, the black region indicates that the chromosome is homozygous for YJM789-derived SNPs. Such patterns for all of sectored colonies are shown in Dataset S1, and the coordinates for each transition are in Dataset S2.

Based on whether the gene conversion tracts contained a 4:0 region, we concluded that 38 of 45 events induced by 20 mM H_2_O_2_ were a consequence of a DSBs in unreplicated chromosomes, and 17 were the consequence of a DSB in a replicated chromatid. Of those 28 events induced by 0.5 mM H_2_O_2_, 17 were associated with formation of a DSB in G_1_. Thus, about three-quarters of the crossovers reflected repair of DSBs in unreplicated chromosomes. From Dataset S2, we also calculated the median length of conversions associated with crossovers on IV as 15.2 kb (95% confidence limits [CL] of 13.8–23.8 kb) for events induced by 20 mM H_2_O_2_ and 11.3 kb (10.1–18.3 kb) for events induced by 0.5 mM H_2_O_2_. Combining both datasets for the hydrogen peroxide-treated samples, the median conversion tract length was 13 kb. These tract lengths are similar to that observed for spontaneous crossovers (10.7 kb) (12). In a previous study (12), we mapped spontaneous recombination events on the right arm of IV, identifying several hotspots (Supplementary Figure S2A); by statistical tests (described in Supplementary Information), only HS4 was significantly ‘hot.’ No significant hotspots were observed for the H_2_O_2_-induced crossovers (Supplementary Figure S2B).

#### Unselected genomic alterations throughout the genome

Fourteen of the sectored colonies (treated with 20 mM H_2_O_2_) were also analyzed using whole-genome SNP arrays, allowing us to detect unselected changes throughout the genome. In addition to the 14 crossover events that occurred on IV, we observed 101 chromosomal alterations (7.2 changes/isolate), including 61 gene conversions unassociated with crossovers, 26 crossovers, 4 BIR events and 3 terminal deletions. In addition, we found seven short terminal LOH events (near the left telomeres of chromosomes I, VIII, XI and XII, and near the right telomeres of chromosomes IV, VI, and XIII). Since these LOH events occurred near the telomere, we were unable to determine whether they represented conversions associated or unassociated with crossovers. The types of changes and the coordinates of LOH transitions for these events are in Dataset S3.

In agreement with our analysis of selected events on chromosome IV, about 85% of the gene conversion events (74 of 87) have 4:0 regions, indicating that the initiating recombinogenic lesion is a DSB on an unreplicated chromosome. The tract lengths of gene conversion events associated and unassociated with crossovers were 13.4 kb (12.5–33.4 kb, 95% CL) and 12.9 kb (13.0–20.8 kb, 95% CL), respectively. We also calculated the proportion of conversion events that are associated with crossovers. Following a mitotic crossover, only half of the segregation patterns result in reciprocal LOH (26). Thus, the observed number of crossovers of 26 should be corrected to 52, and 26 events should be subtracted from the observed numbers of conversion events unassociated with crossovers (61–26 = 35). Although this calculation indicates that more than half of the conversion events are associated with crossovers, the number of conversion events unassociated with crossovers may be slightly underestimated (discussed further below), since small conversion events (<2 kb) may be undetected because they may not include a SNP within the LOH region. In summary, these results, in agreement with our previous conclusions (27), indicate that mitotic conversions with and without associated crossovers are approximately equally frequent. It should be pointed out that in studies in which the length of the conversion tract is limited by the selection system (for example, conversion between repeated genes on non-homologous chromosomes), a smaller fraction of conversions are associated with crossovers. For example, in a study of ectopic recombination by Inbar and Kupiec (28), only 13% of the conversions were crossover-associated.

There were more than ten times more reciprocal crossovers than BIR events. A 6:1 ratio in favor of crossovers was observed previously in UV-treated cells (29). These data confirm previous evidence (24) that crossovers are more common than BIR events in wild-type cells.

### Physical evidence for DSB formation in G_1_-synchonized cells treated with H_2_O_2_

**Figure 4.**
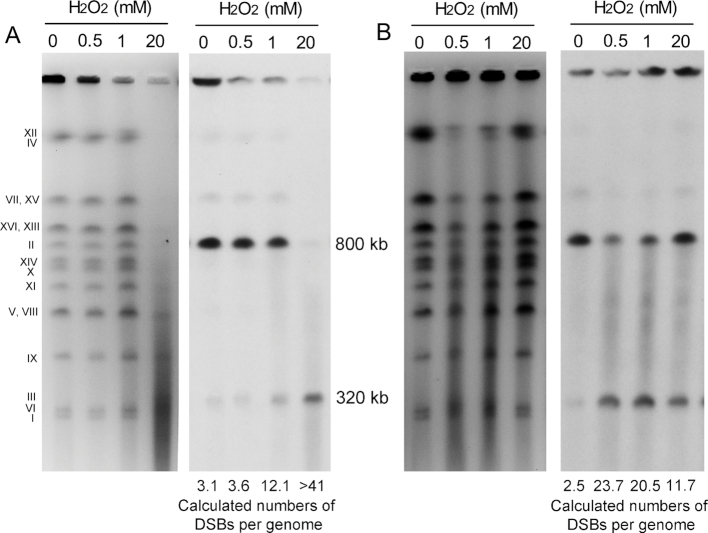
Detection of H_2_O_2_ induced chromosome breaks by gel electrophoresis. The strain used in this study (YYy123) had a circular derivative of chromosome III and a *LEU2* insertion on both III and chromosome II. (**A**) Cells were treated with various concentrations of H_2_O_2_ (0–20 mM), and examined by CHEF gels as described in the Materials and Methods section. The left panel shows the ethidium bromide-stained gel with Roman numerals indicating the approximate locations of various yeast chromosomes. Following electrophoresis, the chromosomal DNA was transferred to membranes and hybridized to a *LEU2*-specific probe (right panel). (**B**) Cell-free samples of DNA embedded in agarose plugs were treated with for one hour with various concentrations of H_2_O_2_, and then examined by gel electrophoresis as in A. The numbers at the bottom of the gel are the calculated numbers of DSBs per genome.

The results described above are strong evidence that the majority of the recombinogenic lesions are DSBs. To confirm this conclusion, we measured DSBs using gel electrophoresis. We used a haploid strain YYy123 that is a *MAT***a** derivative of MWJ50 previously used for a similar purpose by Ma *et al.* (30). This strain has a circular derivative of chromosome III generated by a fusion of *HML* and *HMR*, and an insertion of *LEU2* on both the circular chromosome III and on the linear chromosome II. G_1-_synchronized cells were treated for one hour with 0, 0.5, 1 or 20 mM H_2_O_2_, and then analyzed by clamped-homogenous electric field (CHEF) gel electrophoresis, followed by Southern analysis using a *LEU2*-specific probe. In CHEF gels, large circular chromosomes remain in the gel wells whereas linear chromosomes enter the gel (30). In the left panel of Figure 4A, the ethidium bromide-stained gel is shown; not all of the chromosomes are represented by single bands. On the right panel of Figure 4A, the Southern blot with a *LEU2*-specific probe is shown. Three bands of hybridization are observed: hybridization in the well (representing circular III molecules or replicating molecules), a band at ∼800 kb (linear chromosome II), and a band at about 320 kb (linear III). From the left panel of Figure 4A, it is evident that the highest concentration of H_2_O_2_ results in loss of most of the chromosomes, presumably as a consequence of a high level of DSBs. In the right panel of Figure 4A, the amount of *LEU2*-specific DNA within the well decreases with increased H_2_O_2_, and the amount of hybridization at the position of linear chromosome III increases, as expected. Using the ratio of *LEU2*-specific hybridization for chromosome II and the linear chromosome III, one can calculate the approximate number of genomic DSBs (30). These estimates are given below the gel in the right panel. Hayashi and Umezu (10) previously showed that H_2_O_2_ could induce DSBs in yeast cells. At the same concentrations of H_2_O_2_, the numbers of DSBs appeared considerably more in our study than in their analysis, possibly because of the difference in DSB formation in G_1_-synchronized versus asynchronous cells.

We also demonstrated that H_2_O_2_ can directly break cell-free chromosomal DNA (Figure 4B). DNA in agarose plugs was treated for one hour with various concentrations of H_2_O_2_ before analyzing the samples by gel electrophoresis. The estimated numbers of DSBs/genome (average of three experiments) are shown at the bottom of the right panel of Figure 4B. At the highest concentration of H_2_O_2_, the number of DSBs was reduced relative to some of lower concentrations. Although this result is surprising, in a previous study of *in vitro* nicking of DNA by H_2_O_2_, Luo *et al.* (31) found more nicking at a concentration of 0.05 mM H_2_O_2_ than at concentrations of 50 mM H_2_O_2_. One possible explanation of this result is that H_2_O_2_ may react with ·OH (the most reactive oxygen species) to produce the less reactive oxygen species ·HO_2_ (31). We also point out that it is difficult to compare the *in vivo* and *in vitro* effects of H_2_O_2_, since the various amounts and types of reactive oxygen species generated by H_2_O_2_*in vivo* are affected by the intracellular concentration of iron, and the presence of protective enzymes such as catalases and superoxide dismutases. The main points concerning the data shown in Figure 4A are that the levels of DSBs observed *in vivo* is correlated with the concentration of H_2_O_2_, and that DSBs can also be produced by exposure of cell-free DNA to H_2_O_2_.

### Genomic alterations in yeast cultures exposed to multiple cycles of H_2_O_2_

We also examined the effects of H_2_O_2_ on asynchronous yeast cultures. Colonies were grown by culturing cells on rich solid medium at 30°C for 48 h. Individual colonies were then treated with 100 mM H_2_O_2_ in liquid rich medium for 1 h, followed by plating the cells on solid rich medium. This treatment resulted in ∼30% killing, and a frequency of red/white sectored colonies of 8.9 × 10^−3^ (CL 4–14 × 10^−3^). This frequency of sectoring was about four-fold less than observed by treating G_1_-synchronized cells with 20 mM H_2_O_2_ (Figure 2B). We also examined four of these sectored colonies with whole-genome microarrays, and only one unselected event was identified. This frequency was ∼20-fold less than that observed in G_1_-synchronized cells.

To determine whether the genome-destabilizing effects of H_2_O_2_ were dependent on the 8-OxoG-DNA glycosylase Ogg1p, we also examined the rate of red/white sectored colonies in a diploid strain (SY53) that was homozygous for the *ogg1* mutation. A treatment of asynchronous cells for one hour with 100 mM H_2_O_2_ resulted in a rate of 10^−2^ (CL 5–15 × 10^−3^) of sectored colonies, similar to that observed in the wild-type strain. The frequency of killing was about 40%, also similar to wild-type. One interpretation of this result (to be discussed further below) is that the recombinogenic effects of H_2_O_2_ are not a consequence of the action of the base excision machinery on H_2_O_2_-damaged bases.

Since a single treatment of asynchronous wild-type cells did not result in many unselected events, we treated 30 colonies of JSC25-1 with 100 mM H_2_O_2_ for 20 cycles of sub-culturing as described above, and examined these colonies using whole-genome SNP arrays. A total of 347 genomic alterations (average of 11.6/strain) were observed among these strains that were labeled HOS1-HOS30. We found 226 conversions (interstitial LOH) and 105 terminal LOH events (crossover/BIR) (depicted in Dataset S4 with coordinates given in Dataset S5). The median size of the conversion tracts was 9.9 kb (11.5–15.1, 95% CL). Although our analysis of unsectored strains does not allow us to distinguish whether a terminal LOH event is a consequence of a crossover or BIR event, based on our analysis of sectored colonies, it is likely that most of the terminal LOH events reflect crossovers. The extent of gene conversion tracts associated with crossovers is most readily determined by analysis of both sectors of sectored colonies as shown in Figure 3. However, if the unsectored colony has an interstitial LOH event derived from one homolog adjacent or close to a terminal LOH event derived from the opposite homolog, this colony likely represents a crossover associated with a 4:0 conversion event such as depicted in the bottom part of Figure 3E. In Dataset S5, such events are described as ‘CON/CO’ (conversions associated with crossovers); as described above, such events likely reflect the repair of two broken sister chromatids (Figure 1B).

We also found ten deletions and duplications (Datasets S6 and S7). About half (4 of 10) of these events were interstitial and half (6 of 10) were terminal. Although these events were not characterized in detail, based on our previous studies (13,32), most of the interstitial events likely result from unequal crossovers between Ty elements or other repeated genes. Similarly, the terminal duplication or deletion events are likely to reflect strains in which a DSB occurred within a repetitive element that was repaired by a BIR event on a different chromosome (32). Lastly, we observed five aneuploid isolates: trisomy of chromosomes VII, III and VI in isolates HOS5, HOS25 and HOS30, respectively; and monosomy of chromosomes III in isolates HOS15 and HOS17. In HOS7, the W303-1A-derived copy of XII was lost and there were two copies of the YJM789-derived copy of XII; such events are termed ‘uniparental disomy’ (UPD).

**Figure 5.**
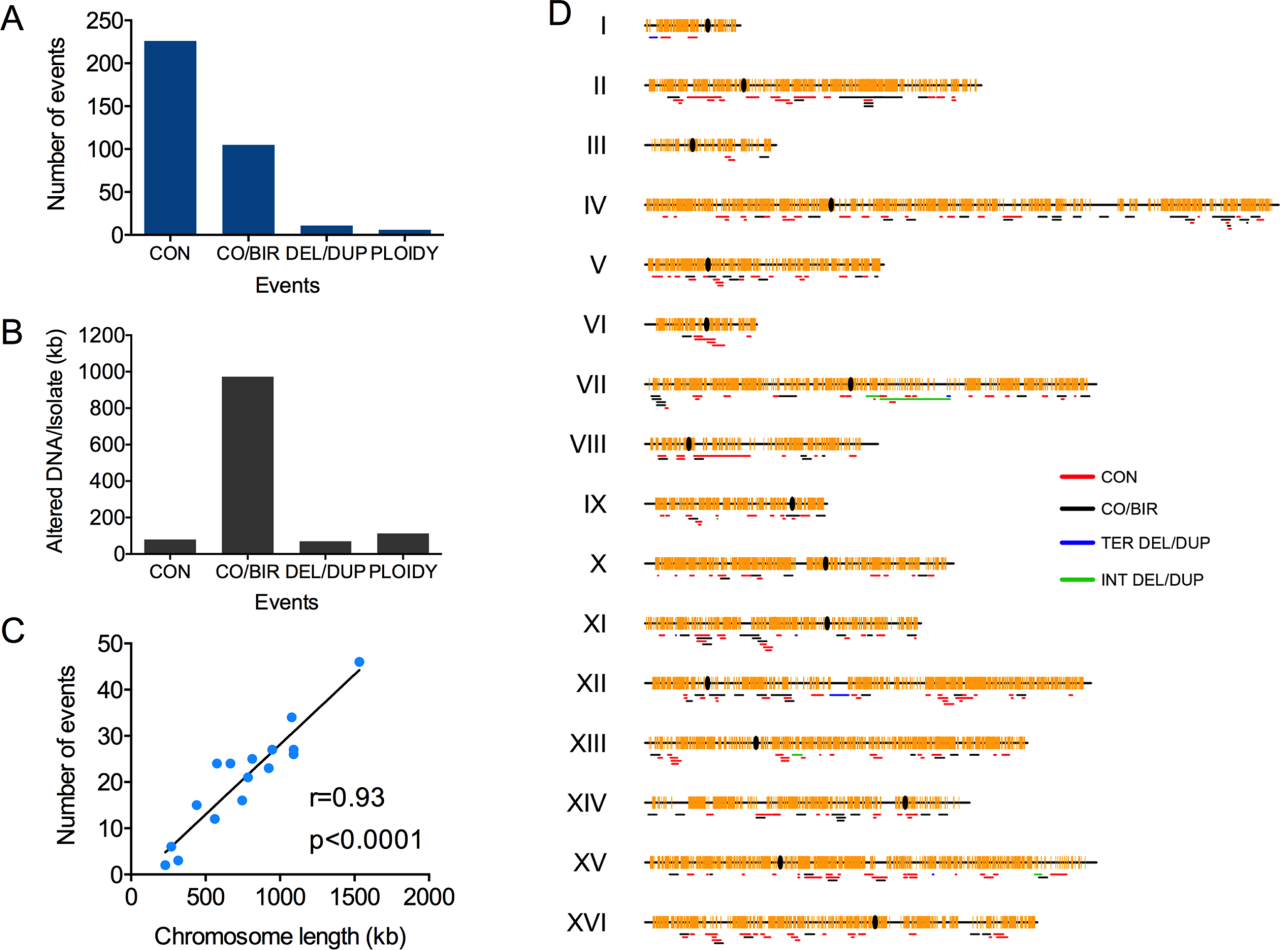
Characterization of genomic alterations induced by multiple treatments with H_2_O_2_ in non-synchronized cells by using whole-genome SNP microarrays. As described in the text, following 20 treatments with H_2_O_2_, we examined 30 independent isolates using whole-genome microarrays. (**A**) Numbers of mitotic recombination and aneuploidy events among the 30 isolates. CON, CO/BIR, DEL/DUP, and PLOIDY designations signify conversions unassociated with crossovers, crossovers or BIR events (which cannot be distinguished except in sectored colonies), deletions or duplications, and changes in chromosome number, respectively. (**B**) Amount of DNA (in kb) per isolate that experienced LOH, or a change in gene dosage for various categories of genomic alterations. (**C**) Number of events per chromosome (sum of categories shown in A) as a function of chromosome length. (**D**) Distribution of LOH and duplication/deletion events across the yeast genome. In this figure, we show interstitial deletion/duplication and terminal deletion/duplication events separately. Centromeres are shown as black ovals, and SNPs are shown as yellow vertical lines.

A summary of the genetic alterations in the 30 isolates is shown in Figure 5A. Based on these data, we also calculated the numbers of kb that were affected per sub-cultured isolate either by LOH or alterations in copy number (Figure 5B). About 9% of the genomes of the isolates underwent LOH, and ∼2% of the genome underwent copy number variations due to chromosome aneuploidy. We also found that the number of events per chromosome was roughly proportional to chromosome length (Figure 5C).

The distribution of recombination events along the chromosomes is shown in Figure 5D. We did not observe any strong hotspots for H_2_O_2_-induced events. We also calculated whether certain chromosomal elements or sequence motifs (such as Ty elements, centromeres, long terminal repeat (LTR) sequences, G4 quadruplex motifs, etc.) are enriched or underrepresented within conversion tracts or near crossover breakpoints (Supplementary Table S3). The details of this type of analysis are described in our previous studies (33,34). None of these chromosomal elements were enriched at the breakpoints. In contrast, there was a significant under-representation of LTRs (*P* = 0.004), tandemly-repeated sequences (*P* = 0.002), and regions of high-GC content (*P* = 0.003); these *P* values remain significant after corrections for multiple comparisons. The last two correlations likely reflect a reduced rate of events in the ribosomal RNA genes, since these genes represent most of the tandem repeats of the genome and have a high-GC content (44%) relative to average genomic GC-content (39%). The reduced rates of homolog recombination in the ribosomal DNA could be an indication of a lower level of oxidative DNA damage in these genes or more efficient repair of oxidative damage by mechanisms (such as base excision) that do not lead to exchange between the two homologs. In addition, although gene conversion events unassociated with crossovers would be undetectable in the ribosomal RNA genes because these genes are represented in >100 copies per array, crossovers are also under-represented. Based on the fraction of the genome that is ribosomal DNA (rDNA) and the observed 105 terminal LOH events, we expected ∼8 in the rDNA. We found none (*P* < 0.01).

### Whole-genome sequencing of H_2_O_2_-treated isolates

To find small genomic alterations that would be undetectable by microarrays, we sequenced 5 out of the 30 JSC25-1-derived isolates (HOS2, HOS5, HOS20, HOS24, and HOS29) by Illumina paired-end high-throughput technology. All 65 genetic alterations detected previously by microarrays were verified by DNA sequencing. In addition, we detected 14 new gene conversion events (Dataset S8). The conversion tract sizes (median size of 1.4 kb) were considerably smaller than those observed by microarrays, as expected, since sequencing identifies LOH events at all 55 000 polymorphisms and the microarrays examine only 15,000 of these SNPs (11). Among the five sequenced isolates, we detected 135 single-base changes and 6 small in/dels (Dataset S9). We checked seven of the single-base mutations and three of the in/dels by PCR and Sanger sequencing, and all were confirmed.

From previous studies (35), the numbers of spontaneous single-base changes and small in/dels expected after 20 cycles of sub-culturing (about 500 generations) of a wild-type diploid are about seven and none, respectively. Thus, most of the alterations detected in our analysis were H_2_O_2_-induced. It is not possible to determine a rate of mutagenesis, since the cells were not grown in medium containing H_2_O_2_. The proportions of the six types of single-base substitutions relative to wild-type are shown in Supplementary Figure S3. The distribution of the six types of changes is significantly different for spontaneous alterations and H_2_O_2_-induced alterations (chi-square test, *P* = 0.02). The interpretation of this result will be discussed further below.

### Analysis of an isolate of JSC25-1 (obtained after repeated exposures to H_2_O_2_) that had increased resistance to H_2_O_2_

**Figure 6.**
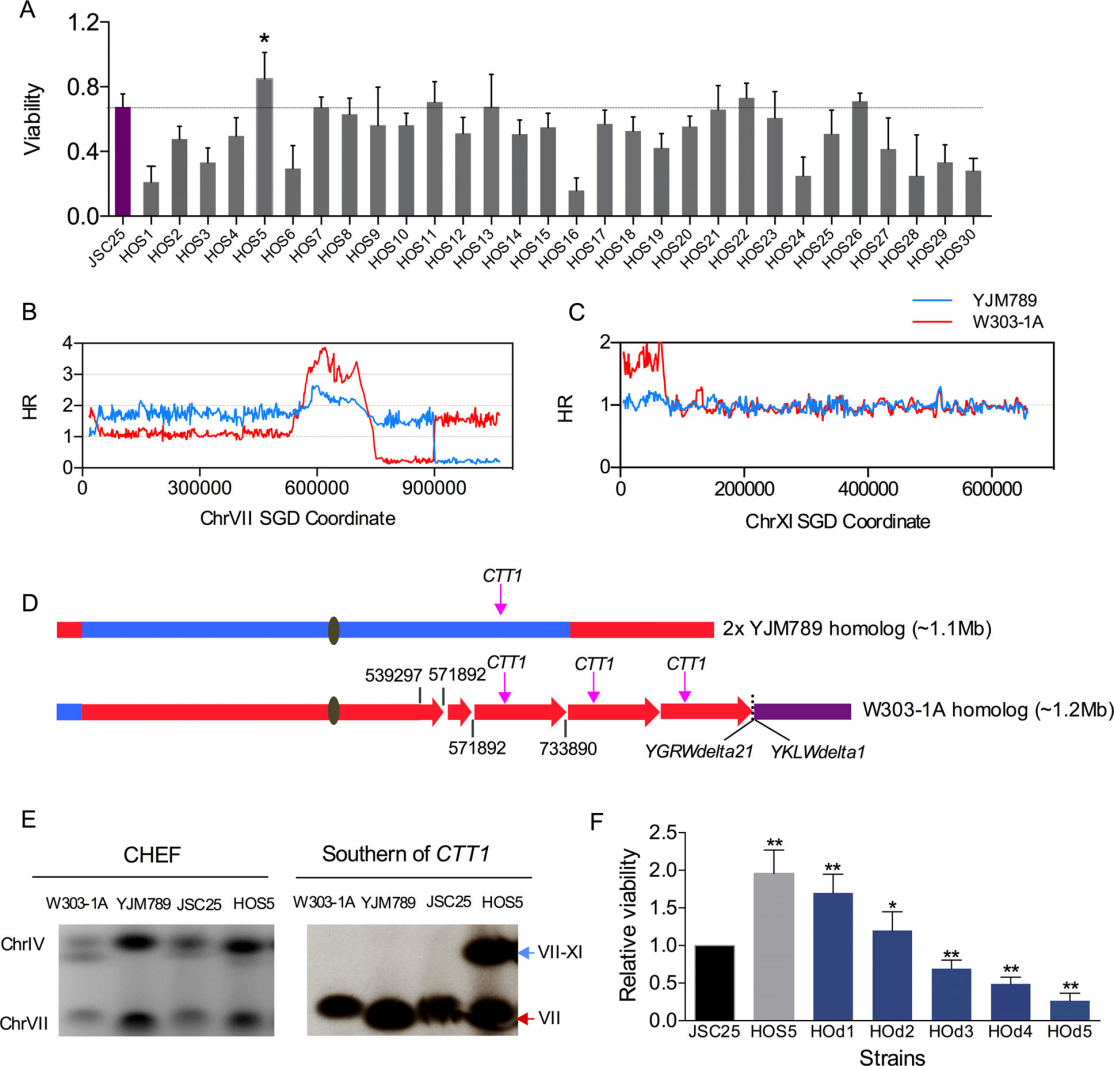
Chromosomal alterations leading to H_2_O_2_ tolerance in JSC25-1 isolates exposed to multiple cycles of H_2_O_2_. (**A**) The H_2_O_2_-tolerance of JSC25-1 and JSC25-1-derived isolates obtained after 20 generations of subculture was measured as described in the text. We used SNP microarrays to examine genomic changes in the most tolerant strain (HOS5). 95% confidence limits on each viability measurement are shown. An asterisk indicates that the viability for the HOS5 strain is significantly (*P* < 0.05) greater than that of the wild-type strain by a *t*-test. (**B**) Patterns of gene dosage and LOH on chromosome VII. The hybridization ratio indicates that HOS5 had three copies of chromosome VII. Two had the same size as the wild-type copy of VII and contained primarily YJM789-derived SNPs. The third copy had multiple internal duplications, had lost sequences distal to SGD coordinate 733890, and had acquired sequences from chromosome XI. (**C**) This microarray shows a region of XI that was transferred to chromosome VII, likely by a BIR event. (**D**) Depictions of the chromosomes that contain VII-derived sequences in HOS5. Red and blue indicate that the sequences were derived from W303-1A and YJM789, respectively. Purple shows the region derived from chromosome XI. The lengths of the various segments are not drawn to scale. (**E**) Gel analysis and Southern blot analysis of JSC25, the parental haploid strains W303-1A and YJM789, and HOS5. The probe used for the Southern hybridization was *CTT1*. (**F**) Resistance of yeast cells to killing by H_2_O_2_ is a function of the number of copies of *CTT1*. We show the viability of cells (normalized to the wild-type diploid JSC25) exposed to 200 mM H_2_O_2_ for 1 h. The *CTT1* copy numbers for the various isogenic strains are: 2 (JSC25), 5 (HOS5), 4 (HOd1), 3 (HOd2), 2 (HOd3), 1 (HOd4) and 0 (HOd5). By *t*-tests, the viability of strains HOS5, HOd1, and HOd2 were significantly greater than for the wild-type strain JSC25 (single asterisks and double asterisks, indicating *P* values < 0.05 and 0.01, respectively). The viabilities of strains HOd3, HOd4, and HOd5 were significantly less than JSC25.

Following exposure of 30 JSC25-1 isolates to 20 exposures to H_2_O_2_ (as described above), we tested them for their sensitivity to H_2_O_2_. Cells were treated with 100 mM H_2_O_2_ for 1 h, and then plated on rich medium in the absence of H_2_O_2_. Compared to the parental JSC25-1 strain, 22 out of 30 strains were less tolerant to H_2_O_2_, whereas four strains showed improved tolerance (Figure 6A). By a *t*-test, the only isolate with significantly elevated tolerance was HOS5, whose survival was about one-third higher than that of the parental strain. By microarray analysis of HOS5, this isolate had ten gene conversions, four crossover/BIR events, four chromosomal deletion/duplication events, and one aneuploid chromosome (Datasets S5 and S7).

Based on microarray analysis, it is evident that chromosome VII in HOS5 had undergone multiple genetic rearrangements (Figure 6B), and sequences derived from the left end of chromosome XI were present in three copies (Figure 6C). The strain had two copies of VII of approximately the normal size, 1100 kb. In these two copies, most of the sequences were derived from YJM789, however, there were two segments of W303-1A-derived sequences (one from the left telomere to coordinate 36 kb and the other from the right telomere to position 905 kb) (upper chromosome in Figure 6D). The third copy of VII had YJM789 sequences on the left end (left telomere to coordinate 36 kb), a duplication of the region between coordinates 539–572 kb, a triplication of the region between coordinates 572–734 kb, and loss of chromosome VII sequences distal to coordinate 734 kb. In addition, about 74 kb of sequences from the left arm of XI were added to this chromosome (bottom part of Figure 6D). The expected length of this homolog is about 1.2 Mb, and a chromosome of approximately this size that hybridized to probes derived from chromosomes VII (Figure 6E) and XI was observed by CHEF gel analysis. As expected, the 1.2 Mb chromosome was observed only in HOS5, and not in JSC25-1 or the parental strains used to construct JSC25-1.

Although the exact pathway producing the complex rearrangement of the homolog with the duplications on chromosome VII is not clear, one scenario is shown in Supplementary Figure S4. The homolog that contains predominantly YJM789 sequences can be generated by two separate recombination events with the W303-1A-derived homolog (likely in different cell divisions), followed by non-disjunction. The other homolog had a number of tandem repeats. Such events could result from repeated cycles of BIR and template switching. These types of duplications usually have repetitive DNA elements (often Ty or delta elements) at the breakpoints of the duplication, but sometimes occur in regions of very limited homology, reflecting microhomology-mediated BIR (MM-BIR) (36,37). Although there are annotated repeated elements at some of the breakpoints in the chromosome VII rearrangement, not all of the breakpoints have such elements. Therefore, the model shown in Supplementary Figure S4 is only tentative, and could involve both BIR and MM-BIR.

As a consequence of the intrachromosomal duplications on one homolog and disomy of the other homolog, the region located between coordinates 540–570 kb on chromosome VII is found in four copies and the region between coordinates 572–739 kb is present in five copies instead of the two copies expected for the wild-type diploid. Among the 65 genes located within the larger 162 kb repeat is the *CTT1* gene that encodes the cytosolic catalase T protein. Catalases break down H_2_O_2_ into dioxygen (O_2_^−^) and water, and *ctt1* cells grown in rich medium are sensitive to H_2_O_2_ (38). Overexpression of *CTT1* results in resistance of wild-type yeast cells to H_2_O_2_ (39).

To confirm the phenotypic effects of gene *CTT1* duplication in HOS5 (five copies of *CTT1*), we made an isogenic deletion series of strains derived from HOS5 that had four, three, two, one or zero *CTT1* genes (details in Supplemental data). The tolerance of the resulting strains to 200 mM H_2_O_2_ was determined. The resistance to killing by H_2_O_2_ of HOS5 was about two-fold higher than that of JSC25-1 (two *CTT1* copies) (Figure 6F). In the other strains, the H_2_O_2_ tolerance was a function of the number of *CTT1* copies. Thus, exposure of wild-type strains to H_2_O_2_ can yield genetic alterations that produce increased H_2_O_2_ tolerance.

### Contribution of endogenous metabolic oxidative stress to spontaneous mitotic recombination

Because *Saccharomyces cerevisiae* can grow in the presence or absence of oxygen, we could examine the effect of endogenous metabolic oxidative stress on the rate of spontaneous mitotic recombination. A diploid strain DZ5 was constructed with a *URA3* gene inserted near the right end of chromosome IV about 700 kb from the centromere (33); this strain is isogenic (except for changes introduced by transformation) with the other diploids described above (Supplementary Table S1). 5-FOA^R^ derivatives of similar strains reflect recombination events (crossovers or BIR events) or chromosome loss (33). Recombination events with breakpoints between the *URA3* insertion and the centromere can be distinguished from chromosome loss events because the latter event results in LOH for markers on both arms of chromosome IV.

**Figure 7.**
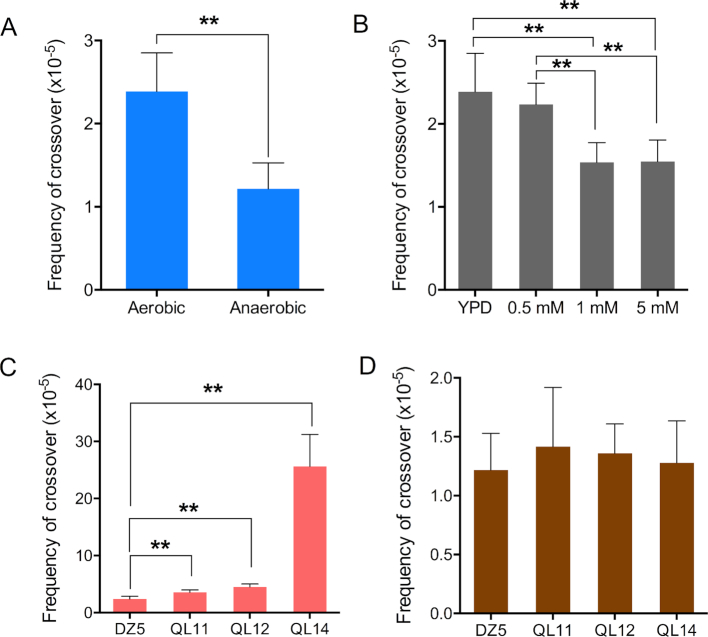
Contribution of oxidative stress to spontaneous mitotic recombination. The frequency of crossovers between *CEN4* and *URA3* on chromosome IV was measured by determining the frequency of 5-FOA-resistant colonies for strain DZ5 and isogenic derivatives. Bars show 95% confidence limits. In comparisons of these values (by *t*-tests), single asterisks and double asterisks indicate *P* values less than 0.05 and 0.01, respectively. (**A**) Growth of yeast under anaerobic conditions resulted in a two-fold reduction of spontaneous crossovers in DZ5 relative to cells grown aerobically. (**B**) Addition of glutathione to the growth medium (YPD) reduced the frequency of spontaneous crossovers. (**C**) Frequency of crossovers in wild-type (DZ5), *ctt1* (QL11), *cta1* (QL12), and *sod1* (QL14) strains grown aerobically. (**D**) Frequency of crossovers in the same strains examined in C, but grown anaerobically. None of the frequencies in Figure 7D were significantly from that observed for DZ5 or from each other.

To examine the effect of oxygen on the rate of mitotic recombination, we streaked cells on rich growth medium, and incubated the plates under aerobic (standard) or anaerobic conditions for three days; anaerobic conditions were achieved using BD GasPacks (Materials and Methods). The resulting colonies (20 colonies/experiment, three experiments) were plated on solid medium containing 5-FOA and on non-selective medium. The rates of 5-FOA^R^ isolates were determined as described in Materials and Methods. Since all isolates (20 of 20) examined by PCR (details in Materials and Methods) were heterozygous for markers on the left arm of IV, we consider that the rate of 5-FOA^R^ derivatives reflects the frequency of recombination rather than chromosome loss, although we cannot distinguish between crossovers and BIR events. As shown in Figure 7A, anaerobic growth reduced the frequency of spontaneous recombination events by 2-fold, from 2.4 × 10^−5^ (CL 1.9–2.9) to 1.2 × 10^−5^ (CL 0.9–1.5).

There are two plausible interpretations of the observation of the results shown in Figure 7A: oxidative damage during aerobic growth is responsible for about half of spontaneous recombination events, or recombinogenic lesions are less efficiently repaired in cells grown anaerobically. To distinguish between these possibilities, we examined the efficiency of repair of a DSB induced in a strain (SJR4317) with the galactose-inducible gene encoding the mega-endonuclease I-SceI (40) under aerobic and anaerobic conditions. This diploid is closely related to JSC25-1 described above, and contains the heterozygous *SUP4-o* marker near the end of the right arm of IV. In addition, the strain contains an I-SceI site on chromosome IV on the right arm of chromosome IV located ∼900 kb centromere-proximal to *SUP4-o*. Thus, when cells are grown on galactose, there is a high rate of red/white sectored colonies as a consequence of recombinational repair of the I-Sce I-generated DSB. We induced DSBs by incubating SJR4317 in medium containing 2% galactose and 2% raffinose for 90 min. The culture was then split and grown aerobically or anaerobically in medium lacking galactose. In the aerobic and anaerobic cultures, the numbers of sectored colonies divided by the total number of colonies were 197/39417 and 118/36403, respectively. By chi-square analysis, the numbers of sectored colonies in the aerobically-grown culture was significantly greater (*P* = 0.002) than in the anaerobically-grown culture. The frequency of sectored colonies in the aerobically-grown culture (5 × 10^−3^, 95% CL of 4.4–5.7 × 10^−3^) is about 50% more than the frequency in the anaerobically-grown culture (3.2 × 10^−3^, 95% CL of 2.7–3.8 × 10^−3^).

Although the two-fold reduction in sectors for spontaneous recombination events in anaerobically-grown cells is slightly greater than the 1.5-fold reduction in repair capacity, the difference is too subtle to prove that oxidative damage in aerobically-grown cells contributes to spontaneous recombination events. Consequently, we did two other types of experiments relevant to this issue. First, using the diploid DZ5 described above, we showed that addition of the ROS-scavenging glutathione to the growth medium reduced the rate of spontaneous crossovers (Figure 7B). Rates were 2.4 × 10^−5^ (CL 1.9–2.9 × 10^−5^), 1.5 × 10^−5^ (CL 1.3–1.8 × 10^−5^), 1,6 × 10^−5^ (CL 1.3–1.8 × 10^−5^) in untreated cells and cells treated with 1 mM or 5 mM glutathione (Figure 7B). Second, we constructed isogenic derivatives of DZ5 lacking *CTT1* (QL11), *CTA1* (QL12), or *SOD1* (QL14) (Figure 7C); *CTA1* and *SOD1* encode catalase A and cytosolic superoxide dismutase, respectively. When cells were grown under aerobic conditions, the rates of crossovers were elevated in all three mutant backgrounds, with the *sod1* mutation having the largest effect (about ten-fold). In contrast, the rates of 5-FOA^R^ in all four strains were similar when the cells were grown in anaerobic conditions (Figure 7D). Taken together, these results argue that oxidative DNA damage may be responsible for initiating about half of spontaneous mitotic recombination events in aerobically-grown cells. However, anaerobically-grown cells also have a partial defect in DSB repair.

## DISCUSSION

H_2_O_2_ is a common metabolic ROS produced in aerobic organisms (7), and is commonly used as a disinfectant. In the experiments described above, we showed that treatment of yeast cells with concentrations of H_2_O_2_ that do not cause great loss of viability are associated with very elevated levels of mitotic recombination between homologs throughout the genome. Many of these events resulted from H_2_O_2_-initiated DSBs in unreplicated chromosomes. Mutations were also induced by H_2_O_2_. Repeated exposure of cells to H_2_O_2_ resulted in an isolate in which the *CTT1* gene was amplified, and we showed that strains with this amplification were more resistant to killing by H_2_O_2_ than wild-type cells. Lastly, we present evidence indicating that environmental oxygen may be responsible for a substantial fraction of spontaneous mitotic recombination events.

### Recombinogenic effects of H_2_O_2_

Most previous studies have shown that H_2_O_2_ treatment elevates mitotic recombination (9,10,41,42). These studies are difficult to compare directly with the current study since recombination was assayed by different methods. For example, Brennan *et al.* (9) found that heteroallelic recombination was elevated ∼10-fold by 2 mM H_2_O_2_ in contrast to the 100-fold elevation observed for crossovers by a similar concentration (2.5 mM) of H_2_O_2_ in our experiments. In a study in which LOH was monitored on the right arm of chromosome III, Hayashi and Umezu (10) found treatment of diploids with 32 mM H_2_O_2_ elevated chromosome loss, crossovers/BIR, and interstitial LOH about 8-, 11- and 29-fold, respectively. Except for chromosome loss rates, the effects are considerably smaller than those observed in our study. In part, this difference may be explained by procedural differences. Most of our quantitative rate measurements were done using cells that were synchronized in G_1_. We observed that 20 mM H_2_O_2_ elevated crossovers about 900-fold in G_1_-synchronized cells, but asynchronous cells treated with 100 mM H_2_O_2_ had only a 200-fold elevation.

Three important questions are: (i) What type of DNA lesion initiates recombination in H_2_O_2_-treated cells? (ii) Is the lesion created by the direct action of ROS on DNA or during the repair of oxidative DNA damage? (iii) Do the number of recombination events correlate with the number of H_2_O_2_-induced DSBs? From many previous studies in yeast and other systems, both DSBs and single-stranded gaps are recombinogenic (8). From our analysis of H_2_O_2_-induced events in G_1_-synchronized cells, more than 80% of the crossovers have the LOH pattern consistent with formation of a DSB on an unreplicated chromosome that was subsequently replicated and repaired in G_2_ (Figure 1B). The remainder had patterns of LOH that could be the consequence of a single broken chromatid (Figure 1A); such a lesion could be the result of a single-stranded nick generated by incomplete base excision repair (BER), followed by replication of the resulting nicked chromosome. It should be emphasized that our method of detecting mitotic recombination events requires an interaction between homologs. Repair of a broken chromatid by sister strand recombination would not lead to LOH and would be undetectable in our experiments.

Two other lines of evidence suggest that most of the hydrogen peroxide-induced recombination events observed in our study are not a consequence of nicks produced by base excision enzymes. First, we find that loss of Ogg1p (involved in the removal of 8-OxoG) does not reduce the level of mitotic crossovers. Second, by gel electrophoresis, we detect a high level of DSBs in both G1-arrested cells and in cell-free DNA (Figure 4). The simplest interpretation of our data is that the reaction of H_2_O_2_ with iron bound to the DNA produces a hydroxyl radical that causes a single-strand nick in the DNA (7). If the repair of this nick is delayed and the iron remains bound, a second nick on the opposite strand would result in a DSB (43).

The last issue to be discussed is the relationship between the numbers of DSBs (measured by gel electrophoresis) versus the numbers of recombination events. For this comparison, we cite the experiments in which the G_1_-synchronized cells were treated with either 0.5 or 20 mM H_2_O_2_. The numbers of DSBs/genome (Figure 4A) were about 0.5 and 40, respectively; for these estimates, we subtract the number of DSBs in untreated cells which likely reflect random breakage of DNA during preparation of the samples. We can estimate the number of recombination events in two ways: by the frequency of sectored colonies and by the frequency of unselected LOH events. The frequencies of red/white sectors in cells treated with 0.5 and 20 mM H_2_O_2_ were 2 × 10^−3^ and 2.8 × 10^−2^, respectively. In our system, red/white sectored colonies result from a crossover between *CEN4* and the *SUP4-o* insertion located 1 Mb from *CEN4*; as pointed out in the Results section, because of the two patterns of chromosome segregation, only half of the crossovers in the *CEN4-SUP4-o* interval result in sectored colonies. This interval is about 8% of the genome. Without correcting for cell viability, the estimated numbers of crossovers/genome for cells treated with 0.5 and 20 mM H_2_O_2_ are 4.2 × 10^−2^ and 7.3 × 10^−1^, respectively. However, since formation of a sectored colony requires that the treated cells produce two viable daughter cells, this calculation needs to be corrected for the loss of viability in the treated cells. For the cells treated with 0.5 mM H_2_O_2_ (viability about 90%), the correction is relatively minor: 1/(0.9)^2^ x 4.2 × 10^−2^ or 5.2 × 10^−2^ events/genome. However, for cells treated with 20 mM H_2_O_2_ (viability about 10%), the correction is major: 1/(0.1)^2^ x 7.3 × 10^−1^ or 73 crossovers/genome. A final correction is that about half of DSBs are repaired by gene conversion events that are unassociated with crossovers. In summary, the calculated number of recombination events in cells treated with 0.5 mM H_2_O_2_ is less than expected from the number of DSBs (0.1 events/genome versus 0.5 DSBs/genome), and the number of events in cells treated with 20 mM H_2_O_2_ is more than expected from the DSB frequency (146 events versus 40 DSBs). Given the many factors used in the calculations, this discrepancy is probably not significant.

We also analyzed by microarrays unselected LOH events throughout the genome in strains treated with 20 mM H_2_O_2_ for one hour. An average of about 7 unselected events was observed per strain (14 isolates examined). This number is smaller than that predicted by the number of DSBs (40 DSBs). Since a different calculation suggests an excess of recombination events, we do not consider this discrepancy significant.

### Mutagenic effects of H_2_O_2_

Although it is clear the yeast strain with mutations in genes that reduce ROS levels or in genes that remove oxidation-damaged bases have elevated levels of mutations (44), there are few studies that examine the mutagenic effects of H_2_O_2_. In studies in mammalian cells, H_2_O_2_ stimulated mutation frequencies 3- to 4-fold (45,46), whereas (as described below) a 10-fold stimulation was observed in yeast (42). One plausible mechanism of mutagenesis is that the unusual pairing properties of some of the oxidized bases would result in misincorporation of the wrong base during replication. For example, one of the most common oxidized bases is 8-oxo-G that can pair with A, leading to a G to T mutation (47). In our experiments, this type of mutation was not enriched in the H_2_O_2_-treated cells, presumably because of the efficient removal of 8-oxo-G by the Ogg1 protein in wild-type cells; loss of Ogg1p results in an elevated level of G to T alterations (48).

In a previous yeast study, Degtyareva *et al.* (42) looked at the mutagenic effects of H_2_O_2_ using two types of strains, one in which the *CAN1* reporter gene was located near the middle of the chromosome (double-stranded DNA reporter) and one in which the C*AN1* gene was located near the telomere (single-stranded DNA reporter). The strain in which *CAN1* was near the telomere had a temperature-sensitive mutation in *cdc13* that resulted in long single-stranded regions at the telomere that included the reporter gene. The results obtained with these two different reporters were strikingly different. The mutation rate in the single-stranded reporter was >100-fold higher in the absence of exogenous DNA damage, and was elevated by only 2-fold by 5 mM H_2_O_2_. Mutations in *REV3*, encoding the error-prone DNA polymerase zeta, reduced the level of H_2_O_2_-induced mutations by only 2-fold. The major class of mutations for the single-stranded reporter was alterations of C, and the rate of these mutations was independent of Rev3p. One interpretation of this result (42) is that, on single-stranded DNA, hydrogen peroxide induces damage of C that, when copied by replicative DNA polymerases, produces a mutation. In contrast, for the double-stranded reporter, H_2_O_2_ treatment induced mutations in the wild-type strain about ten-fold, and most (∼85%) of these mutations were dependent on Rev3p. Although no mutation spectra were done in strains with the double-stranded reporter treated with H_2_O_2_, Northam *et al.* (49) showed that Rev3p-dependent spontaneous mutations had an over-representation of GC to CG, and AT to TA mutations. Thus, the mechanisms by which DNA polymerase zeta produces mutations in single-stranded or double-stranded templates are likely to be different.

In our study, the mutagenic effects of H_2_O_2_ observed in our experiments can be explained two different ways. Since there is a high rate of mitotic recombination in H_2_O_2_-treated cells and since broken DNA ends are processed to generate single-stranded regions of several kb, it is possible that the mutagenesis observed in our experiments could be similar to that observed by Degtyareva *et al.* (42) for the single-stranded reporter (described above). However, since the proportion of C to T and G to A mutations in our experiments is smaller than the fraction observed in untreated cells (0.24 versus 0.35), this mechanism is unlikely. We favor the alternative possibility that the elevated mutation rate in H_2_O_2_-treated cells reflects bases damaged by H_2_O_2_ or by-products of H_2_O_2_ in double-stranded DNA that directly or indirectly recruit DNA polymerase zeta. In support of this possibility, in our mutation spectrum, we found a significant over-representation of AT to TA changes (*P* = 0.01) and an increase (although not statistically significant) in GC to CG changes; these types of alterations are similar to Rev3p-dependent alterations observed by Northam *et al.* (49).

### Evolution of an H_2_O_2_-tolerant strain

Because of the high level of genetic instability associated with H_2_O_2_ treatment, it is not surprising that variants with increased resistance to H_2_O_2_ can be generated. Such variants could reflect *de novo* mutations, LOH events in which an H_2_O_2_-resistant allele becomes homozygous, or a chromosome rearrangement that results in duplication of a gene relevant to systems that protect the cell from oxidative damage. We investigated only one of the H_2_O_2_-resistant isolates, and showed that the causal genetic alteration was an amplification of the *CTT1* gene.

H_2_O_2_-resistant yeast strains can be generated by a number of other genetic alterations. Linder *et al.* (50) showed that duplication of chromosome IV made yeast strains more resistant to H_2_O_2_ as a consequence of duplicating the gene encoding the thioredoxin peroxidase Tsa2p. In mammalian cells, chronic exposure of human fibroblasts to either H_2_O_2_ or 95% O_2_ results in amplification of the *CAT* (catalase) gene, and improved resistance to oxidative stress (51–53). Since there are many genes involved in regulating the cellular response to oxidative stress and since H_2_O_2_ results in a high rate of many types of genomic changes, we expect that resistance to H_2_O_2_ will be generated by a very wide variety of genetic events.

### Source of DNA lesions that lead to spontaneous recombination events

Although most meiotic recombination events are induced by DSBs generated by Spo11p, the DNA lesion responsible for the much-less-frequent spontaneous mitotic crossovers is less clear. The current view is that both DSBs and single-stranded nicks or gaps can produce mitotic exchanges (8). Based partly on the observation that Rad52p foci are much more common in S-phase cells than in cells in other parts of the cell cycle, it was suggested that most spontaneous events were induced during DNA replication. One complication with this conclusion is the finding that about two-thirds of spontaneous crossovers have the gene conversion pattern consistent with a G_1_-initiated event (Figure 1B). A simple resolution of these conflicting conclusions is that most recombinogenic lesions that occur in the S-period are repaired by sister-chromatid recombination, whereas the events that are initiated in G_1_ are repaired by inter-homolog exchange (12); since the two sister chromatids would be broken at the same position in a G_1_-initiated event, an inter-homolog recombination is the only possible pathway for repair by homologous recombination. It should also be noted that inter-sister exchange is 10- to 20-fold more frequent than inter-homolog recombination (54,55).

A number of arguments support the conclusion that oxidative DNA damage is likely one source of recombinogenic lesions responsible for spontaneous events. First, as shown above, H_2_O_2_ treatment is extremely recombinogenic, generating DSBs. Second, elevated levels of ROS are produced by a number of environmental conditions including heat shock, low pH, and osmotic shock (4,56,57). Third, strains with mutations that result in elevated levels of ROS have higher rates of genomic alterations (our results and those of Huang and Kolodner, (58)). Fourth, yeast strains grown in the absence of oxygen have a reduced frequency of spontaneous crossovers relative to aerobically-grown strains. Although this effect is partly a consequence of a reduced efficiency of DSB repair, the observation that growth of cells in the ROS-scavenger glutathione also reduces crossovers is consistent with the suggestion that about half of spontaneous inter-homolog exchanges are a consequence of oxidative DNA damage.

### Summary

Our results argue that DNA lesions resulting from oxidative stress may be general factor that drives genome instability in aerobic cells. We also found that concentrations of H_2_O_2_ that are much lower (50-fold) than those used in commercially-available disinfectants are potent inducers of genetic instability in yeast.

## DATA AVAILABILITY

The accession numbers for the microarrays used in this study are: GSE107178 (whole-genome microarrays of sub-cultured strains) and GSE106816 (whole-genome microarrays and chromosome IV-specific microarrays of sectored colonies). Whole genome sequencing data (clear reads) were deposited in SRA database at NCBI with accession number SRP136146.

## Supplementary Material

Supplementary Data
