# Supplementary material for: Genome-wide analysis of genomic alterations induced by oxidative DNA damage in yeast
Source: Nucleic Acids Res. 2019 Jan 22;47(7):3521–35. doi: 10.1093/nar/gkz027 (PMC6468167; doi:10.1093/nar/gkz027)
Supplement: Supplementary Data [file gkz027_supplemental_files.zip › Supp. Info. 12.11.18.pdf]

SUPPLEMENTAL INFORMATION FOR:

Genome-wide analysis of genomic alterations induced by oxidative DNA damage in yeast

Ke Zhang[1,a], Dao-Qiong Zheng[2,3,a,*], Yang Sui[2,3], Lei Qi[2], and Thomas D. Petes[3,*]

[1]College of Life Science, Zhejiang University, Hangzhou, China 310058

[2]Ocean College, Zhejiang University, Zhoushan, China 316021

[3]Department of Molecular Genetics and Microbiology, Duke University School of Medicine, Durham, NC 27710, USA

[a]These authors contributed equally to this work.

*To whom correspondence should be addressed. Tel: +1 919 684 4986; Fax: +1 919 684 6033; Email: tom.petes@duke.edu. Correspondence may also be addressed to D-QZ. Email: zhengdaoqiong@zju.edu.cn.

SUPPLEMENTAL TEXT

Strain constructions

The genotypes of yeast strains used in this study were listed in Table S1 and the primers used for strain construction were shown in Table S2. The diploid strain JSC25-1 is a hybrid derived by crosses of haploids isogenic with W303-1A and YJM789 (1). JSC25-1 is heterozygous for all SNPs that distinguish W303-1A and YJM789 except for a small region (SGD coordinates 369892-373127) on chromosome XV that is homozygous for YJM789-derived SNPs. JSC25-1 is incapable of undergoing meiosis because of the *MAT*α deletion; it can be synchronized in G_1 using *α*-factor. As a control for the SNP microarray analysis, we used genomic DNA isolated from the diploid strain JSC24-2 that is very similar to JSC25-1 but it does not have the LOH region on chromosome XV (1).

DZ5 is a diploid strain constructed by mating YJM842 and W303HU (2). This strain is heterozygous for a *URA3* insertion near the right end of chromosome IV (Table S1). To delete genes *CTT1*, *CTA1*, and *SOD1*, we amplified DNA from the plasmid pUG6 (3) using primers dCTT1S and dCTT1A, dCTA1S and dCTA1A, and dSOD1S and dSOD1A, respectively. The resulting PCR fragments were transformed into YJM842 and W303HU, selecting for resistance to the drug G418. Deletion of *CTT1*, *CTA1*, and *SOD1* in YJM842 resulted in mutant strains YJMCTT, YJMCTA, and YJMSOD, respectively (Table S1). Similarly, deletion of *CTT1*, *CTA1*, and *SOD1* in W303-1A resulted in strains W303CTT, W303CTA, and W303SOD, respectively. QL11 was obtained by mating YJMCTT and W303CTT. QL12 was constructed by crossing YJMCTA and W303CTA. Mating of YJMSOD1 and W303SOD1 generated QL14.

Strains SY47 and SY48 were obtained by deleting *OGG1* in JSC21-1 and JSC12-1, respectively (Table S1). The cassette used for replacing gene *OGG1* with *kanMX* (construction of SY47). In SY48, *OGG1* was replaced with *URA3* using the PCR fragment produced by amplifying pUG72 DNA with the primers dOGG1US and dOGG1UA. Deletions of *OGG1* were

confirmed by PCR analysis using the primers described in Table S2. The diploid SY49 was constructed by crossing SY47 and SY48. We subsequently replaced the *MATα* gene of SY49 with the *hyg* gene using a PCR-generated fragment obtained by amplifying genomic DNA from the strain JSC25-1 with primers dMATS and dMATA. The resulting strain SY53 was used to determine the effect of *OGG1* on mitotic recombination when yeast cells were treated with H_2O_2.

To delete the *CTT1* genes in the JSC25-1-derived isolate HOS5, we used the primers dCTT1S2 and dCTT1A2 to amplify sequences from the plasmid pUG72 that contains the *Kluyveromyces lactis URA3* gene flanked by *loxP* sites (3). The resulting strain was then transformed with the plasmid pSH65 that contains a galactose-inducible *CRE* gene with the *ble*^{R} (resistance to bleomycin) as the selectable marker (3). Following removal of the *URA3* marker, we selected derivatives that lost the pSH65 plasmid, and repeated the process to serially remove the other *CTT1* genes. The HOS5 derivatives with decreased copy numbers of *CTT1* were named HOd1-HOd5 (Table S1).

Cell synchronization and H_2O_2 treatment

Yeast cells were grown in 7 ml of rich growth medium (YPD) overnight to an OD_{600} of about 0.2. Cells ($5×10^7$) were collected by centrifugation and arrested in G_1 in 1 ml YPD containing 2.5 μg of α-factor. After two hours at 30° C, H_2O_2 was added to the cell culture, and cells were incubated at 30° C for another hour. The cells were then washed twice to remove α-factor and H_2O_2 before plating.

Description of gene conversion and crossover classes

In Dataset S1, we depict various classes of H_2O_2-induced recombination events that were observed in the sectored colonies with a crossover on chromosome IV. Two types of events are shown: events involving the selected crossovers on the right arm of IV and unselected events on other chromosomes (crossovers and gene conversions unassociated with

crossovers). As in our previous studies, each event is shown as a pair of lines (labeled 1 and 2), each line representing one sector. The segments colored green, red, and black indicate that the segment is heterozygous, homozygous for W303-1A-derived SNPs, or heterozygous for YJM789-derived SNPs, respectively. Most of these events can be explained as outcomes of the repair of single-chromatid breaks (SCBs) or double-sister-chromatid breaks (DSCBs). In general, the distinction between SCB and DSCB is based on whether the gene conversion event is of the 3:1 class (3 of the 4 chromosomes in the two sectors derived from one parental haploid and 1 of the 4 derived from the other; SCB) or of the 4:0 class (all four chromosomes with a region derived from one of the parental haploids, DSCB).

Most of the events shown in Dataset S1 were observed in our previous studies (1). Classes A-F represent various types of gene conversion events unassociated with crossovers, whereas Classes G-K show crossovers without conversion tracts or crossover-associated conversion. Classes A and B show 3:1, or 4:0 conversion events, respectively. Classes C and D show various types of 3:1/4:0 hybrid conversion tracts. Classes E and F represent conversion tracts that are interrupted by a heterozygous region (Class E) or a conversion event that involves two different donors (Class F). Class G events are crossovers with no associated conversion tract or a 3:1 conversion tract, and Class H events are crossovers with conversion tracts that have at least one 4:0 region. Classes I-K have different types of complex conversion events. Some of these events may be associated with failure to repair all mismatches within a heteroduplex, correction of mismatches by restoration-type repair instead of conversion-type repair or branch migration of the Holliday junction intermediate (1,4).

Class L events have the patterns of LOH suggestive of break-induced replication (BIR). These classes are consistent with a SCB in which the broken fragment centromere-distal to the break is lost and the fragment proximal to the break invades and duplicates sequences derived from the other homolog. In most of the Class M events, there is a terminal 4:0 LOH

event. This pattern could be explained as a DSCB event in which two terminal regions from one homolog were lost, followed by two BIR events.

Associations of chromosomal elements with recombination breakpoints

As in previous studies (2,5), we determined whether there was a significant association between recombination breakpoints and various chromosome elements (centromeres, origins of replication, tRNA genes, and other elements listed in Table S3). For each recombination event in the strains treated with multiple cycles of H_2O_2, we determined a “window” that contained the recombination breakpoint (Column J of Dataset S5). This window included sequences between the left marker of the leftmost LOH transition and the right marker of the rightmost LOH transition. We determined the number of elements observed within these windows and outside of these windows for the complete dataset. For example, we found 96 tRNA genes within these windows, and 8124 tRNA genes that were not located in these regions. Based on the size of the yeast genome, the number of strains examined, the fraction of the genome included within the windows, and the number of elements in the genome, we could calculate an expected number of tRNA genes within and outside of the windows (Table S3). The expected numbers were 115 tRNA genes within the windows and 8105 tRNA genes located outside of the windows. By a chi-square test, the difference between the observed and expected values is not significant (p=0.07). Information about the numbers and locations of the various genomic elements is in Song *et al.* (5) and Zheng *et al.* (2).

Analysis of mitotic recombination hotspots

Figure S2 shows mapping of crossover-associated mitotic gene conversions on the right arm of chromosome IV, both spontaneous events (Fig. S2A) and hydrogen peroxide-induced events (Fig. 2B). As described in the text, microarray analysis of red/white sectored colonies allows detection of conversion events associated with crossovers. Although not all sectored colonies have a detectable region of gene conversion, most do. 121 of the 139 spontaneous

crossovers were associated with a gene conversion (1) and 62 of 73 H_2O_2-induced crossovers had an associated conversion. The median conversion tract lengths for spontaneous and H_2O_2-induced were 10.6 kb and 13 kb, respectively. Since the length of the right arm of IV was 1061 kb, we calculate that the probability that a single SNP will be involved in a conversion event if the distribution of such events is random is 10.6 kb/1061 kb or 0.01 for spontaneous events, and about 13 kb/1061 kb or 0.012 for H_2O_2-induced events.

We used the binomial expansion to calculate the probabilities that a single SNP would be involved no times, once, twice, etc. For example, to calculate the probability that a SNP would be involved three times in examining the 121 conversion-associated crossovers of spontaneous events, we substituted n=121, k=3, p=0.99, and q=0.01 in the equation: $[n!/(n-k)!\ k!]\ p^{n-k} \times q^{k}$ which gives a probability of 0.088. The same type of calculation was done to generate probabilities for the number of times individual SNPs would be included in a conversion tract in 121 samples. The largest number of times a SNP was involved in a conversion event was ten, associated with HS4 (Fig. 2A). The probability of a specific SNP being involved this number of times was calculated to be 4.5×10^{-7}. Since we examined about 2300 SNPs on the right arm of IV, it is necessary to correct probabilities for multiple comparisons. Using the Hochberg and Benjamini method of correction (6), we found that HS4 was significantly hot even after this correction. None of the other hotspots in Fig. S2A were significant, and none of SNP peaks in Fig. S2B were statistically significant.

SUPPLEMETNAL TABLES

Table S1. Yeast strains used in this study.

Strain	Background	Construction	Genotype
JSC25-1	W303-1A/YJM789	St. Charles and Petes (1)	*MAT**a**/MATα::hyg ade2-1/ade2-1 can1-100Δ::natMX4/ CAN1Δ::natMX4 ura3-1/ura3 trp1-1/TRP1 his3-11,15/HIS3 leu2-3,112/LEU2 RAD5/RAD5 IV1510386::kanMX6-can1-100/IVI1510386::SUP4-o GAL2/gal2*
JSC24-2	W303-1A/YJM789	St. Charles and Petes (1)	*MAT**a**/MATα::natMX4 ura3/ura3-1 ade2-1/ade2-1 trp1-1/TRP1 his3-11,15/HIS3 leu2-3,112/LEU2 RAD5/RAD5 IV1510386:kanMX6-can1-100/IVI1510386::SUP4-o GAL2/gal2*
YJM842	YJM789	From John McCusker, Duke University	*MAT**a** ho::hisG his3Δ-200 ura3*; isogenic with YJM789
W303HU	W303-1A	Insert *HIS3* and *URA3* in IV147333 and IV1168918, respectively, in YZ26-2 (2)	*MATα leu2-3,112 his3-11,15 ura3-1 ade2-1 trp1-1 can1-100::natMX4 RAD5 IV1147333::HIS3 IV1168918::URA3*
DZ5	W303-1A/YJM789	Cross of W303HU and YJM842	*MAT**a**/MATα LEU2/leu2-3,112 his3Δ-200/his3-11,15 ura3/ura3-1 ADE2/ade2-1 TRP1/trp1-1 CAN1/can1-100::natMX4 IV1147333/ IV1147333::HIS3 IV1168918/IV1168918::URA3*
YYy123	CG329	From Yi Yin, University of Washington; Dmitry Gordenin, NIEHS	*MAT**a** lys2::Alu-DIR-LEU2-lys2DEL5' ura3-DEL leu2-3,112 his7-2 trp1-289 ade5-1* + circular derivative of chromosome III
YJMCTT	YJM789	Delete *CTT1* in YJM842	*MAT**a** ho::hisG his3Δ-200 ura3 ctt1::kanMX6*
W303CTT	W303-1A	Delete *CTT1* in W303HU	*MATα leu2-3,112 his3-11,15 ura3-1 ade2-1 trp1-1 can1-100::natMX4 RAD5 IV1147333::HIS3 IV1168918::URA3 ctt1::kanMX6*
QL11	W303-1A/YJM789	Cross of YJMCTT and W303CTT	*MAT**a**/*MATα *LEU2/leu2-3,112 his3Δ-200/his3-11,15 ura3/ura3-1 ADE2/ade2-1 TRP1/trp1-1 CAN1/can1-100::natMX4 IV1147333/ IV1147333::HIS3 IV1168918/IV1168918::URA3 ctt1::kanMX6/ ctt1::kanMX6*
YJMCTA	YJM789	Delete *CTA1* in YJM842	*MAT**a** ho::hisG his3Δ-200 ura3 cta1::kanMX6*
W303CTA	W303-1A	Delete *CTA1* in W303HU	*MATα leu2-3,112 his3-11,15 ura3-1 ade2-1 trp1-1 can1-100::natMX4 RAD5 IV1147333::HIS3 IV1168918::URA3 cta1::kanMX6*
QL12	W303-1A/YJM789	Cross of YJMCTA and W303CTA	*MAT**a**/MATα LEU2/leu2-3,112 his3Δ-200/his3-11,15 ura3/ura3-1 ADE2/ade2-1 TRP1/trp1-1 CAN1/can1-100::natMX4 IV1147333/ IV1147333::HIS3 IV1168918/IV1168918::URA3 cta1::kanMX6/ cta1::kanMX6*
YJMSOD1	YJM789	Delete *SOD1* in YJM842	*MAT**a** ho::hisG his3Δ-200 ura3 sod1::kanMX6*
W303SOD1	W303-1A	Delete *SOD1* in	*MATα leu2-3,112 his3-11,15 ura3-1 ade2-1*

		W303HU	*trp1-1 can1-100::natMX4 RAD5 IV1147333::HIS3 IV1168918::URA3 sod1::kanMX6*
QL14	W303-1A/YJM789	Cross of YJMSOD1 and W303SOD1	*MAT***a***/MATα LEU2/leu2-3,112 his3Δ-200/his3-11,15 ura3/ura3-1 ADE2/ade2-1 TRP1/trp1-1 CAN1/can1-100::natMX4 IV1147333/ IV1147333::HIS3 IV1168918/IV1168918::URA3 sod1::kanMX6/ sod1::kanMX6*
HOS5	W303-1A/YJM789	Derivative of JSC25 with five copies of *CTT1* (see main text)	*MAT***a***/MATα::hyg ade2-1/ade2-1 can1-100Δ::natMX4/ CAN1Δ::natMX4 ura3-1/ura3 trp1-1/TRP1 his3-11,15/HIS3 leu2-3,112/LEU2 RAD5/RAD5 IV1510386::kanMX6-can1-100/IVI1510386::SUP4-o GAL2/gal2* 5 copies of *CTT1*
HOd1	W303-1A/YJM789	Transform HOS5 with PCR fragment with *LoxP-URA3*-LoxP, followed by expression of Cre. Derivative of HOS5 with four copies of *CTT1*	*MAT***a***/MATα::hyg ade2-1/ade2-1 can1-100Δ::natMX4/ CAN1Δ::natMX4 ura3-1/ura3 trp1-1/TRP1 his3-11,15/HIS3 leu2-3,112/LEU2 RAD5/RAD5 IV1510386::kanMX6-can1-100/IVI1510386::SUP4-o GAL2/gal2* 4 copies of *CTT1*
HOd2	W303-1A/YJM789	Transform HOd1 with PCR fragment with *LoxP-URA3*-LoxP, followed by expression of Cre. Derivative of HOS5 with three copies of *CTT1*	*MAT***a***/MATα::hyg ade2-1/ade2-1 can1-100Δ::natMX4/ CAN1Δ::natMX4 ura3-1/ura3 trp1-1/TRP1 his3-11,15/HIS3 leu2-3,112/LEU2 RAD5/RAD5 IV1510386::kanMX6-can1-100/IVI1510386::SUP4-o GAL2/gal2* 3 copies of *CTT1*
HOd3	W303-1A/YJM789	Transform HOd2 with PCR fragment with *LoxP-URA3*-LoxP, followed by expression of Cre. Derivative of HOS5 with two copies of *CTT1*	*MAT***a***/MATα::hyg ade2-1/ade2-1 can1-100Δ::natMX4/ CAN1Δ::natMX4 ura3-1/ura3 trp1-1/TRP1 his3-11,15/HIS3 leu2-3,112/LEU2 RAD5/RAD5 IV1510386::kanMX6-can1-100/IVI1510386::SUP4-o GAL2/gal2* 2 copies of *CTT1*
HOd4	W303-1A/YJM789	Transform HOd3 with PCR fragment with *LoxP-URA3*-LoxP, followed by expression of Cre. Derivative of HOS5 with one copy of *CTT1*	*MAT***a***/MATα::hyg ade2-1/ade2-1 can1-100Δ::natMX4/ CAN1Δ::natMX4 ura3-1/ura3 trp1-1/TRP1 his3-11,15/HIS3 leu2-3,112/LEU2 RAD5/RAD5 IV1510386::kanMX6-can1-100/IVI1510386::SUP4-o GAL2/gal2* 1 copy of *CTT1*
HOd5	W303-1A/YJM789	Transform HOd4 with PCR fragment with *LoxP-URA3*-LoxP, followed by expression of Cre. Derivative of HOS5 with no copies of *CTT1*	*MAT***a***/MATα::hyg ade2-1/ade2-1 can1-100Δ::natMX4/ CAN1Δ::natMX4 ura3-1/ura3 trp1-1/TRP1 his3-11,15/HIS3 leu2-3,112/LEU2 RAD5/RAD5 IV1510386::kanMX6-can1-100/IVI1510386::SUP4-o GAL2/gal2* 0 copies of *CTT1*
SJR4317	W303-1A/YJM789	Hum and Jinks-Robertson (7)	*MAT***a***/MATα::URA3 ade2-1/ade2-1 can1-100Δ::natMX4/ CAN1Δ::natMX4 ura3-1/ura3 trp1-1/TRP1 leu2-3,112/LEU2 RAD5/RAD5 IV583534:I-SceI/I-SceInc IV1510386/IVI1510386::SUP4-o GAL2/gal2 his3Δ::pGAL-I-SceI-hph/HIS3*
JSC12-1	W303-1A	St. Charles and Petes (1)	*MAT***a** *leu2-3,112, his3-11,15 ura3-1 ade2-1 trp101 can1-100::natMX4 RAD5*

			IV1510386::kanMX6-can1-100
JSC21-1	YJM789	St. Charles and Petes (1)	*MATα ade2-1 ura3 gal2 ho::hisG CAN1::natMX4 IV1510386::SUP4-o*
SY47	YJM789	Deletion of *OGG1* in JSC21-1	*MATα ade2-1 ura3 gal2 ho::hisG CAN1::natMX4 IV1510386::SUP4-o ogg1::kanMX6*
SY48	W303-1A	Deletion of *OGG1* in JSC12-1	*MAT***a** *leu2-3,112, his3-11,15 ura3-1 ade2-1 trp101 can1-100::natMX4 RAD5 IV1510386::kanMX6-can1-100 ogg1::KlURA3*
SY49	W303-1A/YJM789	Cross of SY47 and SY48	*MAT***a***/MATα ade2-1/ade2-1 can1-100Δ::natMX4/ CAN1Δ::natMX4 ura3-1/ura3 trp1-1/TRP1 his3-11,15/HIS3 leu2-3,112/LEU2 RAD5/RAD5 IV1510386::kanMX6-can1-100/IVI1510386::SUP4-o GAL2/gal2 ogg1::kanMX6/ogg1::KlURA3*
SY53	W303-1A/YJM789	Deletion of *MATα* in SY49	*MAT***a***/MATα::hyg ade2-1/ade2-1 can1-100Δ::natMX4/ CAN1Δ::natMX4 ura3-1/ura3 trp1-1/TRP1 his3-11,15/HIS3 leu2-3,112/LEU2 RAD5/RAD5 IV1510386::kanMX6-can1-100/IVI1510386::SUP4-o GAL2/gal2 ogg1::kanMX6/ogg1::KlURA3*

Table S2. Primers used in strain constructions or strain analyses.

Primer	Sequence	Purpose
dCTA1S	TCTTCACTGTAATGTCCAAATCGTACATTTGAA TTTCTTGTAGGTCGACAACCCTTAAT	Replacement of *CTA1* with *kanMX6*
dCTA1A	GAAACAACGCCACTCATTTGTTACTTGAGCGT TTCAAAATTTGGATCTGATATCACCTA	
vPUG6S	CTTCATTACAGAAACGGCT	Verification of *CTA1* deletion
vCTA1A	GCAAACTCTGTCTCAAAGC	
dCTT1S	TACTCTCTACAAAACGGTTTTCCGTACTCTCA TCACCCATACGCTGCAGGTCGACAACC	Replacement of *CTT1* with *kanMX6*
dCTT1A	AACCTTCAAGGTCAACAGGTTCCCAAGGAACT CCCAAGCATTCTGATATCACCTAATAA	
vCTT1A	CCGATCTAAGTACCGAATT	Paired with vPUG6S to verify *CTT1* deletion
dSOD1S	CGCGCAAACAAATAAAACATAATTAATTTATAA TGGTTCAAGCAGGTCGACAACCCTTA	Replacement of *SOD1* with *kanMX6*
dSOD1A	TAACATTAGTTGGTTAGACCAATGACACCACA GGCTGGTCTTGGATCTGATATCACCTA	
vSOD1A	AGAATGGAGCAACATAAGG	Paired with vPUG6S to verify *SOD1* deletion
dCTT1S2	ATTAAAAAAATCCTTCTCTTGTCTCATGCCAAT AAGATCAATCAGGTCGACAACCCTTAA	Replacement of *CTT1* with *URA3* in HOS5
dCTT1A2	AATAAATAGTGCTGCCTTAATTGGCACTTGCA ATGGACCAAGTCTGATATCACCTAATAA	
vCTT1S	TATTGATGTTGGACGAGTC	Verification of *CTT1* deletion in HOS5 and related HOd strains
vCTT1A2	CTAACGAGTTGTGAAAACC	
7-617164S	CATGAAAGAACCGTTGGAG	To verify the deletion of base A at 617164 on chrVII in HOS25
7-617164A	TCACTACAAACCGAGGGAT	
4-940662S	CTAAGACAGAGGGAACCAT	To verify the deletion of base G at 940662 on chrIV in HOS21
4-940662A	CAGTTAAGATTGCCTTGAG	
15-474660S	CTTCATTATTATCGGTCTTG	To verify the deletion of bases CC at 474660 on chrXV in HOS31
15-474660A	GCATTTGATATTACCTGGT	
3-52680S	CGTATCCCACCGTACTTACC	To verify the C to T mutation at 52680 on chrIII in HOS2
3-52680A	GAAGCCATTCGGACCTTAG	
4-937537S	GAACAGATTTGGTGGGATT	To verify the C to A mutation at 937537 in HOS21
4-937537A	TGCGTAGGAATCTTTGTTC	
7-373617S	TTGGACGATTGTTCCTCTA	To verify the A to G mutation at 373617 on chrVII in HOS25
7-373617A	TGAGATGACGATGGTCAAC	
13-383485S	TAATATCCTCGTGACCTCG	To verify the C to T mutation at 383485 on chrXIII in HOS31
13-383485A	TCATCTAATTGCCTTGCAC	
8-270512S	TTGAGAGACATCTGACCGT	To verify the T to A mutation at 270512 on chrVIII in HOS5
8-270512A	TTATGACTGTGCCAAAGTG	
15-220978S	CACTGCACCATTAAGTCCT	To verify the G to T mutation at 220978 on chrXV in HOS5
15-220978A	GAGATAAAGTGAGCGGAGA	
12-66233S	GCTTCAAATGGCTATTGTC	To verify the T to G mutation at 66233 on chrXII in HOS2
12-66233A	GTCGTGATGCCTTTGTTAG	
vChrIVMonS	AACCTTTAACATTCAGGGAG	Primers used to detect LOH on the left arm of IV
vChrIVMonA	ATGACTGCTTGGTAGTTGAG	
dOGG1S	AATTGCGATTTTATTTATCAACCAGATGTCTTA	Replacement of *OGG1* with

	TAAATTCGGCAGGTCGACAACCCTTA	*kanMX6* in JSC21-1
dOGG1A	TACCACTAGTCCCTCCGATTTCTTTAGAGAAT AGGACACCTTGGATCTGATATCACCTA	
vSYPUG6S	ATGCGTCAATCGTATGTG	Verification of *OGG1* deletion in SY48
vOGG1A	TGTGAGACCACCTATTGAAG	
dOGG1US	AATTGCGATTTTATTTATCAACCAGATGTCTTA TAAATTCGGCAGGTCGACAACCCTTA	Replacement of *OGG1* with *URA3* in JSC12-1
dOGG1UA	AAGATCAGACAATTCAACTTTCAGTTTCATTTG TTTCGTAACAATACGCAAACCGCCTC	
vSYPUG72S	CCATTTGATGCTCGAGGC	Verification of *OGG1* deletion in SY47, paired with primer vOGG1A
OGG1S	ATTTGTTGCTGTCGGCGACTG	Verification of *OGG1* deletion in SY47 and SY48
OGG1A	CTCTCTAACGTCTTCATATTG	
dMATS	AATCGTCCTGTCCCATTACG	Replacement of *MATα* with *HYG*
dMATA	TTGGAAACACCAAGGGAGAG	
MATR	AGTCACATCAAGATCGTTTATGG	Primers used for PCR analysis of mating type
MATαF	GCACGGAATATGGGACTACTTCG	
MATaF	ACTCCACTTCAAGTAAGAGTTTG	

Table S3. Analysis of the associations of various genomic elements with breakpoints of recombination.

Chromosome elements	Predicted # of elements included in microarrays	Observed # within tracts	Observed # outside of tracts	Exp. # within tracts	Exp. # outside of tracts	p value*
tRNA genes	274	96	8124	115	8105	0.068
ARS elements	317	129	9381	134	9376	0.690
snRNA and snoRNA genes	83	35	2455	35	2455	0.997
ncRNA genes	14	8	412	6	414	0.384
Transposable elements	48	17	1423	20	1420	0.470
Solo long-terminal repeats (LTRs)	274	85	8135	115	8105	0.004
Centromeres	16	2	478	7	473	0.066
Palindromic sequences	570	245	16855	240	16860	0.755
G4 sequences (motif 1)	544	238	16082	229	16091	0.560
Highly-transcribed genes	330	153	9747	139	9761	0.234
Weakly-transcribed genes	312	114	9246	131	9229	0.125
Rrm3p pause sites	112	45	3315	47	3313	0.747
Regions with high levels of gamma-H2AX	631	241	18689	266	18664	0.124
Replication-termination (ter) sequences	71	28	2102	30	2100	0.724
Tandemly-repeated sequences	1299	475	38495	547	38423	0.002
Regions of high GC (>42%) content	538	182	15958	227	15913	0.003
Regions of low GC (<35%) content	407	163	12047	172	12038	0.513
Inefficient T-T dimer repair	142	47	4213	60	4200	0.095
Meiotic recombination hotspots	136	66	4014	57	4023	0.248

*Chi-square test performed in Microsoft Excel. The p values shown in red are significant after correction for multiple comparisons.

SUPPLEMENTAL FIGURES

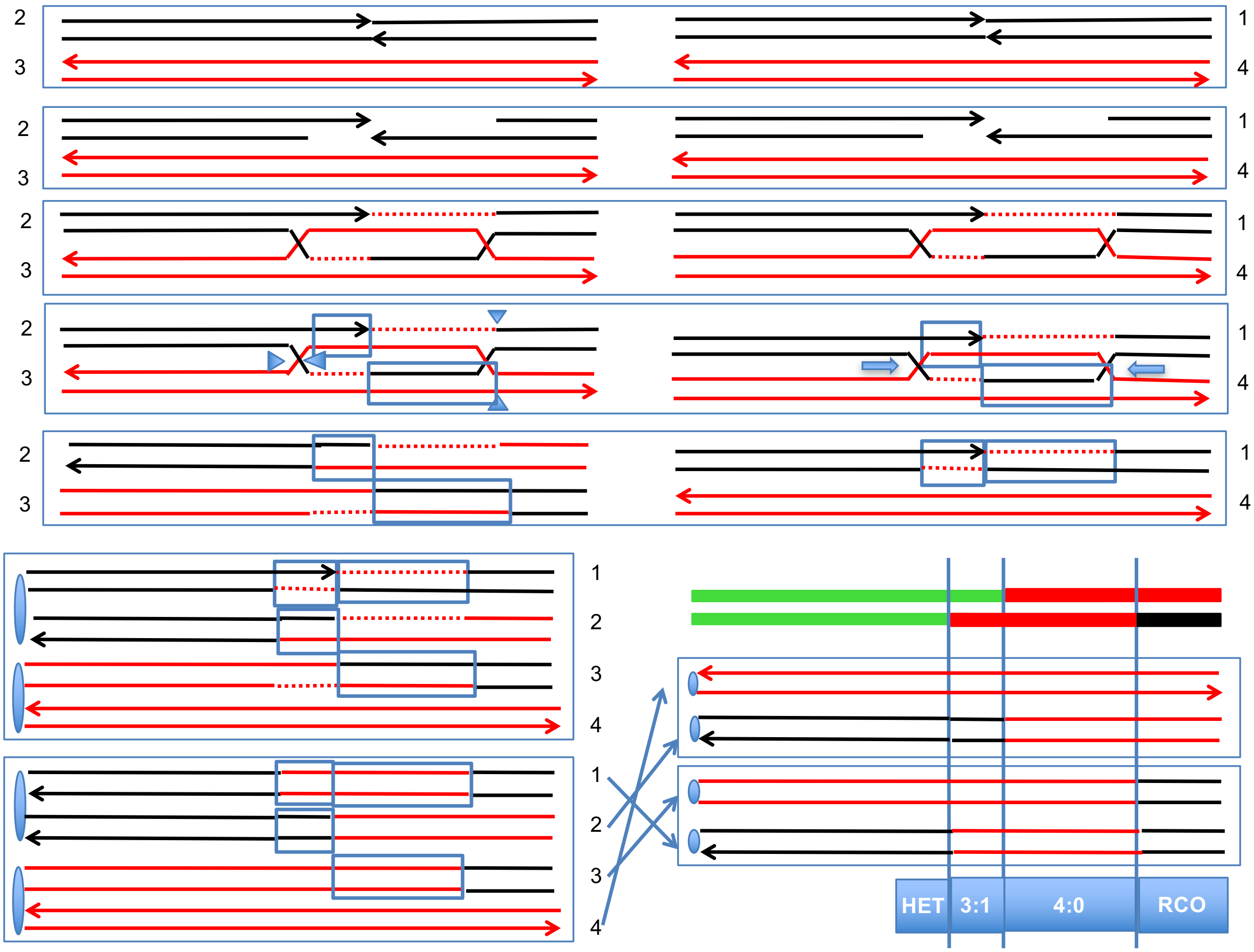

Figure S1. Patterns of heteroduplex formation and mismatch repair that would yield the recombination event shown in Fig. 3 (HO-20-22 in Dataset S2). Chromosomes are shown as double-stranded DNA molecules with terminal arrows indicating the 3′ ends. Red and black lines show sequences from W303-1A- and YJM789-derived homologs, respectively. Dotted lines indicate DNA synthesis, and blue ovals represent centromeres. This crossover event was initiated by a DSB on the YJM789-derived homolog in G_1 phase, resulting in DSBs on chromatids 1 and 2 at the same positions. The broken chromatids pair with the unbroken chromatids, forming two double Holliday junctions (dHJs). Blue boxes show regions of heteroduplexes. Processing of the junctions is indicated by blue triangles. The dHJ involving chromatids 2 and 3 was processed to yield a crossover, whereas the junction involving 1 and 4 was processed as a non-crossover. Mismatches within the heteroduplex regions were

repaired to generate either two red strands or two black strands. The net result of these events is a reciprocal crossover associated with a hybrid 3:1/4:0 conversion tract.

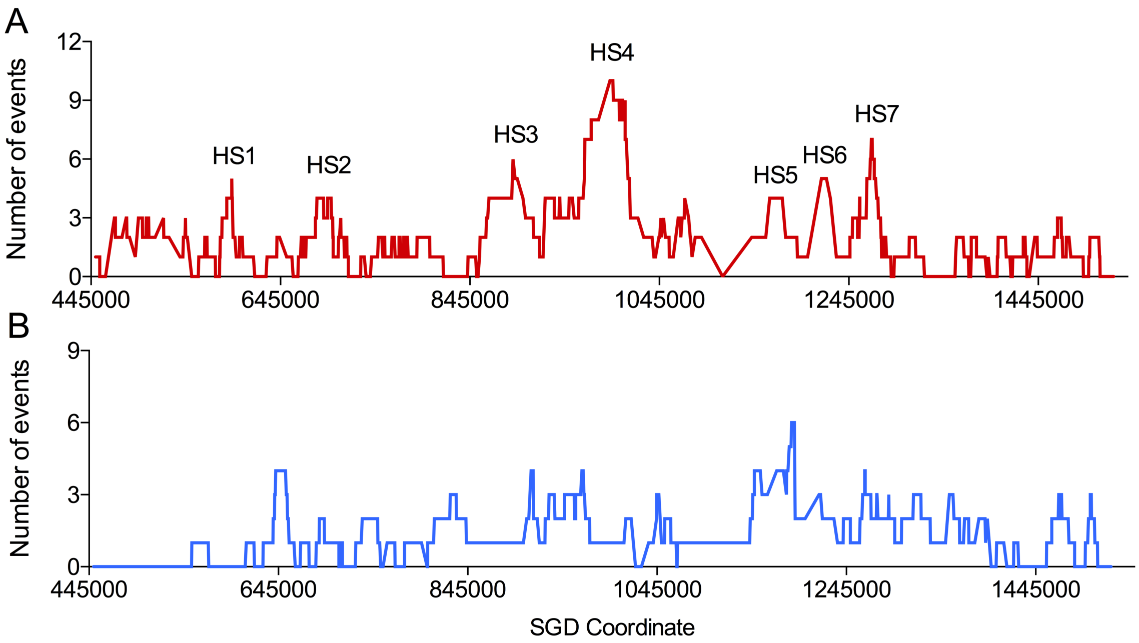

Figure S2. Distributions of spontaneous and H_2O_2-induced crossovers on the right arm of chromosome IV. These plots summarize the number of times a SNP is included in conversion tracts associated with a crossover on chromosome IV. A. Spontaneous crossovers (data derived from ref. 1). Of the recombination hotspots labeled H1-H7, only H4 is significantly hotter than the other regions on the right arm of IV after corrections for multiple comparisons (details of the analysis described in the S1 Text). B. H_2O_2-induced crossovers. None of the peaks reflect significant hotspots. Note that HS4 is missing in the H_2O_2-induced events.

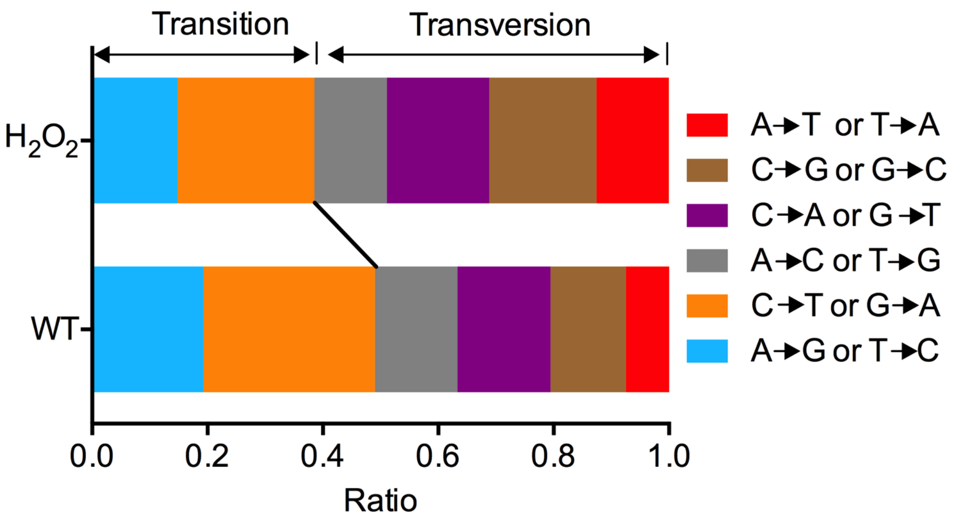

Figure S3. Patterns of H_2O_2-induced single-base substitutions. The proportions of the six possible base substitutions are shown. Data for the wild-type diploid was from Zhu *et al.* (8). The numbers of mutations in the H_2O_2-treated samples and in the untreated strain are 135 and 867, respectively.

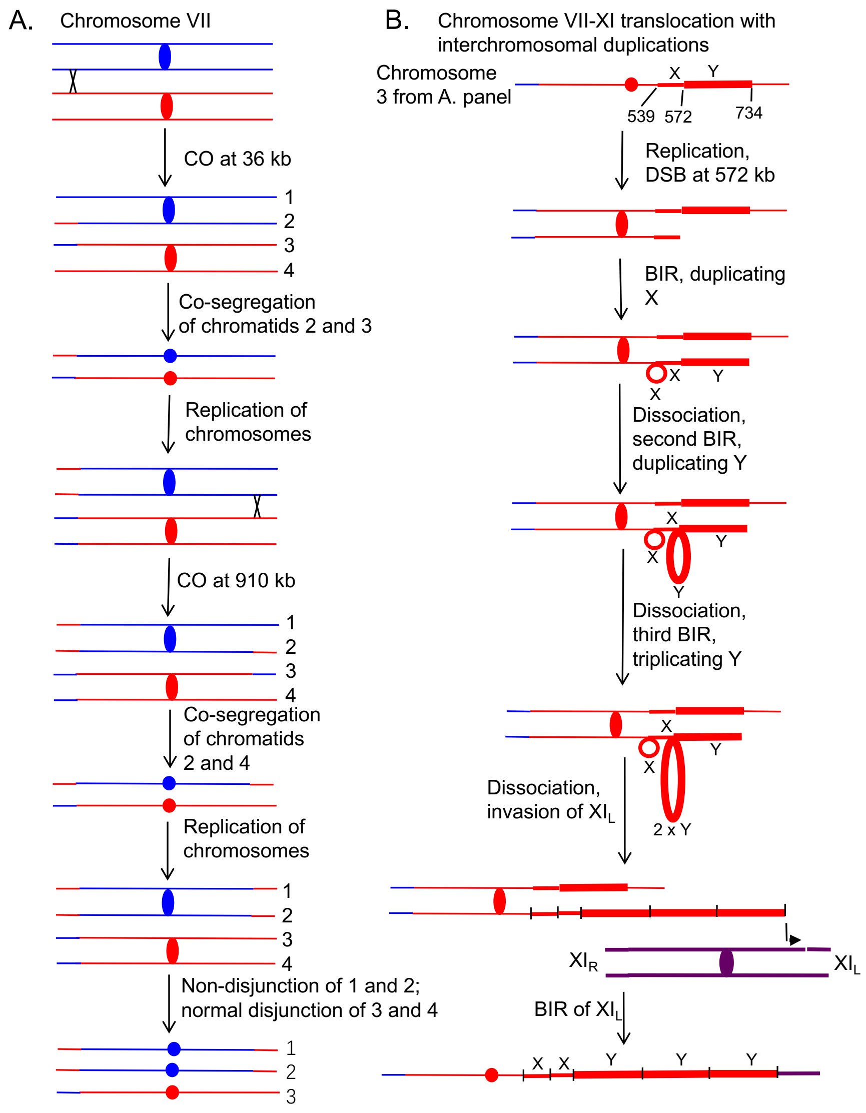

Figure S4. Formation of duplications and translocations by repeated cycles of BIR. The strain HO5 was trisomic for chromosome VII (Fig. 6). Two identical chromosomes contained primarily YJM789-derived SNPs with segments derived from the W303-1A homolog at the left and right ends. The other chromosome was composed primarily of W303-1A SNPs, but had YJM789-derived SNPs at its left and and chromosome XI sequences at its right end. We show the generation of these chromosomes in two steps. A. Inter-homolog recombination followed by non-disjunction. Chromosome VII regions derived from the W303-1A and YJM789 homologs are shown in red and blue, respectively. Centromeres are shown as ovals or circles. These events resulted in trisomy for chromosome XI. The chromosome labeled “3” underwent additional alterations as shown in B. B. Production of

intrachromosomal duplications and a translocation in chromosome “3” shown in A. In this diagram, we show sequences between coordinates 539 and 572 kb (labeled X), and between 572 and 734 kb (labeled Y) as thick lines. Following DSB formation near position 572 kb, the broken end invades the sister chromatid at position 539 kb, resulting in a duplication of X. When the replication fork reaches position 734 kb, the end dissociates and re-invades the sister chromatid at position 572 kb, duplicating Y. When the fork reaches position 734 kb, it dissociates again, re-invading the sister at position 572 kb, resulting in a triplication of Y. Finally, when the fork reaches position 734 kb, it dissociates and the broken end invades the left arm of chromosome XI near coordinate 75 kb. The resulting chromosome has a duplication of X, a triplication of Y, and a segment of chromosome XI at the right end of VII. This pathway is only tentative, and sequence analysis of each junction would be necessary to confirm it.

SUPPLEMENTAL REFERENCES

1. St. Charles, J. and Petes, T.D. (2013) High-resolution mapping of spontaneous mitotic recombination hotspots on the 1.1 Mb arm of yeast chromosome IV. *PLoS Genet.*, **9**, e1003434.
2. Zheng, D.Q., Zhang, K., Wu, X.C., Mieczkowski, P.A. and Petes, T.D. (2016) Global analysis of genomic instability caused by DNA replication stress in *Saccharomyces cerevisiae*. *Proc. Natl. Acad. Sci. U. S. A.*, **113**, E8114-E8121.
3. Gueldener, U., Heinisch, J., Koehler, G.J., Voss, D. and Hegemann, J.H. (2002) A second set of *loxP* marker cassettes for Cre-mediated multiple gene knockouts in budding yeast. *Nucleic Acids Res.*, **30**, e23.
4. Yin, Y. and Petes, T.D. (2013) Genome-wide high-resolution mapping of UV-induced mitotic recombination events in *Saccharomyces cerevisiae*. *PLoS Genet.*, **9**, e1003894.
5. Song, W., Dominska, M., Greenwell, P.W. and Petes, T.D. (2014) Genome-wide high-resolution mapping of chromosome fragile sites in *Saccharomyces cerevisiae*. *Proc. Natl. Acad. Sci. U. S. A.*, **111**, E2210-2218.
6. Hochberg, Y. and Benjamini, Y. (1990) More powerful procedures for multiple significance testing. *Stat. Med.* **9**, 811-818.
7. Hum, Y.F. and Jinks-Robertson, S. (2017) Mitotic gene conversion tracts associated with repair of a defined double-strand break in *Saccharomyces cerevisiae*. *Genetics*, **207,** 115-128.
8. Zhu, Y.O., Siegal, M.L., Hall, D.W. and Petrov, D.A. (2014) Precise estimates of mutation rate and spectrum in yeast. *Proc. Natl. Acad. Sci. U. S. A.*, **111**, E2310-2318.
